# Past climate-driven range shifts structuring intraspecific biodiversity levels of the giant kelp (*Macrocystis pyrifera*) at global scales

**DOI:** 10.1038/s41598-023-38944-7

**Published:** 2023-07-25

**Authors:** Jorge Assis, Filipe Alberto, Erasmo C. Macaya, Nelson Castilho Coelho, Sylvain Faugeron, Gareth A. Pearson, Lydia Ladah, Daniel C. Reed, Peter Raimondi, Andrés Mansilla, Paul Brickle, Giuseppe C. Zuccarello, Ester A. Serrão

**Affiliations:** 1 CCMAR, CIMAR, Universidade do Algarve, Gambelas, Faro, Portugal; 2 Faculty of Bioscience and Aquaculture, Nord Universitet, Bodø, Norway; 3 Department of Biological Sciences, University of Wisconsin, Milwaukee, USA; 4 Centro Fondap IDEAL and Departamento de Oceanografía, Universidad de Concepción, Concepción, Chile; 5 University of Pittsburgh School of Medicine, Pittsburgh, USA; 6 Núcleo Milenio MASH and IRL3614 Evolutionary Biology and Ecology of Algae, Facultad de Ciencias Biológicas, CNRS, Sorbonne Université, Universidad Austral de Chile, Pontificia Universidad Catolica de Chile, Santiago, Chile; 7 Departamento de Oceanografía Biológica, Centro de Investigación Científica y de Educación Superior de Ensenada, Ensenada, Baja California Mexico; 8 Marine Science Institute, University of California Santa Barbara, Santa Barbara, USA; 9 University of California, Santa Cruz, USA; 10 Cape Horn International Center (CHIC), Universidad de Magallanes, Punta Arenas, Chile; 11 South Atlantic Environmental Research Institute, Stanley, Falkland Islands; 12 School of Biological Sciences, Victoria University of Wellington, Wellington, New Zealand

**Keywords:** Marine biology, Population genetics, Climate-change ecology, Ecological modelling, Molecular ecology, Population genetics

## Abstract

The paradigm of past climate-driven range shifts structuring the distribution of marine intraspecific biodiversity lacks replication in biological models exposed to comparable limiting conditions in independent regions. This may lead to confounding effects unlinked to climate drivers. We aim to fill in this gap by asking whether the global distribution of intraspecific biodiversity of giant kelp (*Macrocystis pyrifera*) is explained by past climate changes occurring across the two hemispheres. We compared the species’ population genetic diversity and structure inferred with microsatellite markers, with range shifts and long-term refugial regions predicted with species distribution modelling (SDM) from the last glacial maximum (LGM) to the present. The broad antitropical distribution of *Macrocystis pyrifera* is composed by six significantly differentiated genetic groups, for which current genetic diversity levels match the expectations of past climate changes. Range shifts from the LGM to the present structured low latitude refugial regions where genetic relics with higher and unique diversity were found (particularly in the Channel Islands of California and in Peru), while post-glacial expansions following ~ 40% range contraction explained extensive regions with homogenous reduced diversity. The estimated effect of past climate-driven range shifts was comparable between hemispheres, largely demonstrating that the distribution of intraspecific marine biodiversity can be structured by comparable evolutionary forces across the global ocean. Additionally, the differentiation and endemicity of regional genetic groups, confers high conservation value to these localized intraspecific biodiversity hotspots of giant kelp forests.

## Introduction

The distribution of cold and temperate marine biodiversity at the infraspecific level often carries the footprints of past climate-driven range shifts^1–3^. Particularly well reported are range shifts of low dispersive or sessile species driven by glacial-interglacial cycles of the Quaternary ﻿(c. 2.6 Myr to the present). The advance of ice sheets and compression of isotherms during glacial periods (e.g., Last glacial Maximum; c. 20 Kyr ago)^4,5^ caused poleward range contractions and lower latitude expansions^2,6–9^, while the opposite trend would have happened during interglacials (e.g., Holocene; c. 12 Kyr to the present)^9–13^. Behind such range-edge extinction-recolonization regions, climatic refugia are expected, where populations could have persisted for sufficient time to enable the evolution of genetic hotspots and endemic lineages^7^. In contrast, where extinctions/recolonizations occurred, populations tend to be genetically depauperate and highly homogeneous due to genetic bottlenecks along colonization fronts^7,14–17^. Despite the paradigm of past climate-driven range shifts in structuring intraspecific biodiversity, biological models exposed to comparable conditions in independent geographic regions are rarely available to allow for independent replicated assessment.

The higher latitude regions of the Northern vs. Southern hemispheres provided contrasting evolutionary conditions for marine coastal species, particularly considering the differences in ice extent and shoreline morphology during glacial periods^18^. The Northern hemisphere was buried under thick layers of permanent ice down to the latitudes matching Brittany (France) and Maine (USA) in the Atlantic Ocean, and Vancouver Island (Canada) in the Pacific Ocean^5^. In the Southern hemisphere, besides Antarctica and its surrounding islands, only Patagonia was severely affected^18^, thus providing additional opportunities for population persistence. However, a large portion of the Southern Hemisphere’s coasts are distributed in tropical areas, potentially providing less suitable habitat for cold-temperate species than in the Northern Hemisphere, where the coastline is more continuous from equatorial to Arctic regions. Major shifts in southern oceanic circulation, led by the intensification of the Circumpolar Antarctic Current (ACC) during LGM, likely added complexity to the glacial seascape, even in regions not affected by ice cover. For instance, the strong cooling of the Humboldt Current may have provided suitable habitat even in the inter-tropical zone^19^. Another peculiarity of the Southern hemisphere is the role of trans-oceanic dispersal at the end of LGM, leading to eastward (e.g., from New Zealand to Chile), instead of southward recolonization of many species in Patagonia and other sub-Antarctic regions^20–22^. Numerous studies across the globe have shown the genetic effects of past climate changes^2,10,11,14,23,24^, but comparisons between hemispheres are lacking. ﻿Additionally, while the role of refugia as sources for poleward expansions is well‐documented, the effect of past genetic bottlenecks at lower latitudes is insufficiently understood^8^. Testing the drivers of intraspecific biodiversity on a single model species distributed in both hemispheres reduces potential confounding effects linked to comparing species’ specific responses to climate changes, which can largely result from interspecific niche differentiation^25,26^.

The giant kelp *Macrocystis pyrifera* represents an ideal model to address the topic owing to its broad antitropical distribution^27^, with past distributional edges exposed to replicate conditions of higher (cold) and lower (warm) latitudes of both hemispheres. Additionally, its limited spore dispersal and ability to promote density barriers by local saturation of available habitats^28^ may allow retaining the signatures left by past demographic changes^2,8,10^. Giant kelp can further perform long‐distance dispersal events by rafting^29^, potentially extending distributional ranges and following climate velocity changes across whole biogeographic regions. Most importantly, while describing the drivers of intraspecific biodiversity, the identification of endemic genetic lineages for giant kelp is timely, as regional persistence might be currently threatened by ongoing and projected climate changes^30,31^. The recent increase in frequency and intensity of marine heat wave events has been linked to regional losses of the species in both hemispheres^32,33^, which can erode global diversity levels and shift genetic baselines^34^. These losses can also threaten the multiple ecosystem services provided, such as the role in blue carbon sequestration and coastal protection, as well as the nursery effect for numerous economically important species^27,35–37^.

Previous genetic studies with *Macrocystis pyrifera* were mostly performed without broadscale replication across the hemispheres. These aimed to explore the patterns of the species’ genetic structure^38–41^, disentangle ﻿the effect of quaternary climate change fluctuations in the distribution and productivity of the species^42^, the role of seascape drivers mediating genetic differentiation, by testing the role of isolation by habitat continuity, environment and ocean transport^39,43,44^ , as well as the vulnerability of populations to ongoing climatic stress^45^. Three studies sampling populations across the two hemispheres^46,47^ showed (1) *Macrocystis* as a monospecific genus based on two genetically distinguishable ecomorphs, *M. pyrifera* and *M. integrifolia*, (2) the northern^46–48^ hemisphere distribution as the potential ancestral origin of the genus, with consequently reduced genetic diversity in the southern hemisphere, and (3) highlighted the potential role of ice advances in structuring the distribution of genetic diversity. Yet, studies lacked independent estimates of past climate oscillations (e.g., SDM-based), were mostly performed with traditional mtDNA markers, typically prone to selection and poor resolution^48^, and were limited in sampling design. These together might have limited conclusions on how climate-driven range shifts structured the species’ intraspecific biodiversity of giant kelp.

In this study, we asked whether the distribution of intraspecific marine biodiversity is explained by past climate‐driven range shifts across the hemispheres. We compared the genetic variability of *Macrocystis pyrifera*, estimated across the species’ entire distributional range, with independent Species Distribution Modelling (SDM) predicting past range shifts and long-term refugial regions from the last glacial maximum to the present. Overall, we hypothesize that the exposure to comparable past climate changes in the two hemispheres structured disjunct long-term refugial regions currently displaying higher and unique gene pools, relative to regions more recently colonized (i.e., resulting from post-glacial expansions).

## Methods

The study area comprised the entire distributional range of *Macrocystis pyrifera*, from Alaska to Baja California Sur (Mexico) in the northeast hemisphere, and from Peru to Tierra del Fuego (Argentina), as well as South Africa, Australia, New Zealand and sub-Antarctic Islands in the southern hemisphere.

### Species distribution modelling

Species distribution modelling (SDM) estimated past distributional shifts and potential refugial regions where *M. pyrifera* might have occurred for the long-term. This approach used Boosted Regression Tress (BRT), a machine learning algorithm with high predictive performance, able to fit non-linear relationships and complex interactions, while strongly reducing overfitting through hyper-parametrization and forcing of monotonic responses^49,50^.

Biologically meaningful benthic predictors (i.e., along bottom layers) for the species^29^ were developed^52^ for the present-day and the LGM conditions using the computation pipelines of Bio-ORACLE^51^. These reflected essential resources (nutrients as Phosphate and Nitrate), disturbance (ice thickness) and factors affecting physiology (salinity and temperature). Predictors were clipped to 30 m depth, the typical maximum depth distribution of the species^52^. The LGM predictors considered sea level and shoreline displacements by placing the isobath of − 120 m below current sea level^53^. Presence records were extracted from the fine-tuned dataset of marine forest species^54^. The same number of pseudo-absences as presences were randomly generated in sites where no presences were recorded^55^. To reduce the negative effect of autocorrelation in the models, the correlation of predictors within the range of occurrence records (presence and pseudo-absences) was tested as a function of geographic distance. For this purpose, a correlogram was built to pinpoint the minimum distance at which predictors were significantly correlated. Records were pruned by randomly selecting one record from the pool found within such distance^56^.

﻿Considering the general pattern found in the species’ genetic structure (see “Results”), models were developed separately for the northern and southern hemispheres^57^. A tenfold cross-validation framework using independent latitudinal bands^7^ was implemented to find the optimal hyperparameter combination of number of trees (50–1000, step 50), tree complexity (1–6) and learning rate (0.01, 0.005 and 0.001). This approach also inferred the performance of models^58^ by using the area under the curve (AUC) of the receiver operating characteristic curve and sensitivity^59^. To reduce overfitting, models were forced to fit negative monotonic responses for maximum temperature and ice thickness, and positive responses for nutrients, salinity and minimum temperature^7,49,60^. The relative contribution of predictors to the models was determined by computing the increase in deviance explained (i.e., goodness of fit) when each predictor was added to its alternative model. Physiological tolerance limits (maximum and minimum, depending on the predictor) were estimated from individual response functions produced for each predictor, while fixing all alternative predictors to their averages^61,62^.

Parsimonious models (i.e., with fewer predictors) with higher potential for transferability^63^ were built using a stepwise approach by fitting a full model (i.e., with all predictors) and interactively removing predictors one at the time, from the least to the higher contributive, until the difference of deviance explained between the full model and the reduced model was higher than zero^58,60,64^. Maps reflecting the potential distribution of the species for present-day conditions and the LGM were developed with the parsimonious models fitting the optimal hyperparameters. These maps were reclassified to binomial surfaces, reflecting presence and absence of *Macrocystis pyrifera*, by applying a threshold maximizing both specificity (true negative rate) and sensitivity^7,58,65^. Range shifts were determined in terms of area by comparing the LGM and present modelled distributions for the regions where genetic sampling took place (see “Methods” below). Regions providing glacial refugia were identified where the species was predicted to occur during the LGM period. Because the species can produce rafts that disperse for long periods of time over the surface of the ocean^29^, predictions were also performed for the LGM and the present with surface layers^51^.

### Genetic structure and diversity

Approximately 30 individuals were sampled per site (115 sites; 3872 individuals in total; S1) for genetic analysis, by removing a piece of the blade and preserving it in silica drying crystals. Genomic DNA extraction was performed with the NucleoSpin 96 Plant Kit II (Macherey–Nagel, Duren, Germany) and standard protocols. Microsatellite amplification and scoring was performed for 6 polymorphic loci using PCR conditions and methods described in^66^ and^39^.

Genetic structure between sites was inferred with Structure software^67^ without a priori population assignment, allowing admixture, using the model of correlated allele frequencies for a range of K clusters between 1 and 10, a burn-in time of 2 × 10^5^ repetitions and 1 × 10^6^ iterations. The potential number of clusters was inferred with the DeltaK criterion^68^, which identifies the “best” K value as the one maximizing the rate of change in the log probability of the data (ΔK). The analysis was performed under two hierarchical steps^3,8^: the first aimed to discover the main genetic groups when considering the entire distribution, and the second aimed to reveal the regional levels of genetic structure within each of the main genetic groups that resulted from the first step. This approach allows the analysis of regional genetic differentiation within the main genetic groups independently of their differences in allelic richness. Two sites showing signs of admixture in the first hierarchal step (see “Results”) were excluded from the second step to better disentangle genetic structure^3,8^.

Genetic structure was also inferred with a network analysis, with nodes defined by the sampled sites and edges by their relative number of shared alleles^69^. Excessive network connections with surplus information were removed until a threshold maximizing Modularity^70^. The leading eigenvector algorithm using the percolated network assigned a unique membership (i.e., cluster) to the nodes based on the edge’s distance. The significance of this process was inferred by testing the proportion of 10^4^ random membership assignments retrieving higher Modularity than observed. Network eigenvector centrality was also determined to identify sites serving as hubs to gene flow^69^.

Genetic differentiation was determined between clusters with Jost's D. This index was chosen in detriment of *F*_ST_ because it is more suitable for comparisons with contrasting levels of diversity^71^, as in this case (see “Results”). Genetic diversity per site and cluster was inferred as standardized allelic richness, gene diversity (expected heterozygosity) and private alleles, for the smallest sample size found in any population (excluding sizes ≤ 10) using 10^4^ randomizations.

## Results

### Species distribution modelling

﻿The marine forests dataset retrieved 10,715 occurrence records for the species. Correlograms identified predictors autocorrelated at a minimum distance of 4 km in the northern group, and 9 km in the southern group, pruning a final dataset of 180 and 369 records, respectively.

The distribution models included multiple predictors, from which temperatures (maximum and minimum) had a prominent role in explaining the distribution of both northern and southern genetic groups (combined relative contributions: 95.06% and 75.15%, for the northern and southern hemispheres, respectively; Table 1). Thermal tolerance was estimated between 3.22 and 24.23 °C for the northern group and between 1.94 and 23.98 °C for the southern group. The modelled distribution of the southern group was further dependent on nutrients above ~ 0.02 mmol.m^−3^ and ice thickness above 0.16 m (contributions ranging between 5.69 and 10.63%; Table 1). An additional niche overlap analyses performed between the northern and southern groups (S2) showed evidence of niche conservatism in *Macrocystis pyrifera* (i.e., the northern and southern hemispheres display identical ecological niches).

**Table 1:** Relative contribution of each predictor to the performance of the models (bold values depicting contributions above 5%) and estimates of physiological tolerance limits.

Variable	Relative contribution (%)		Tolerance limit	
	N. hemisphere	S. hemisphere	N. hemisphere	S. hemisphere
Max. temperature	**54.72**	**59.49**	24.23 °C	23.98 °C
Min. temperature	**40.34**	**15.66**	3.22 °C	1.94 °C
Phosphate	1.89	**8.51**	0.01 mmol.m^−3^	0.02 mmol.m^−3^
Nitrate	1.71	**10.63**	0.01 mmol.m^−3^	0.01 mmol.m^−3^
Salinity	0.14	–	13.09	–
Ice thickness	1.17	**5.69**	0 m	0.16 m

**Figure 1:**
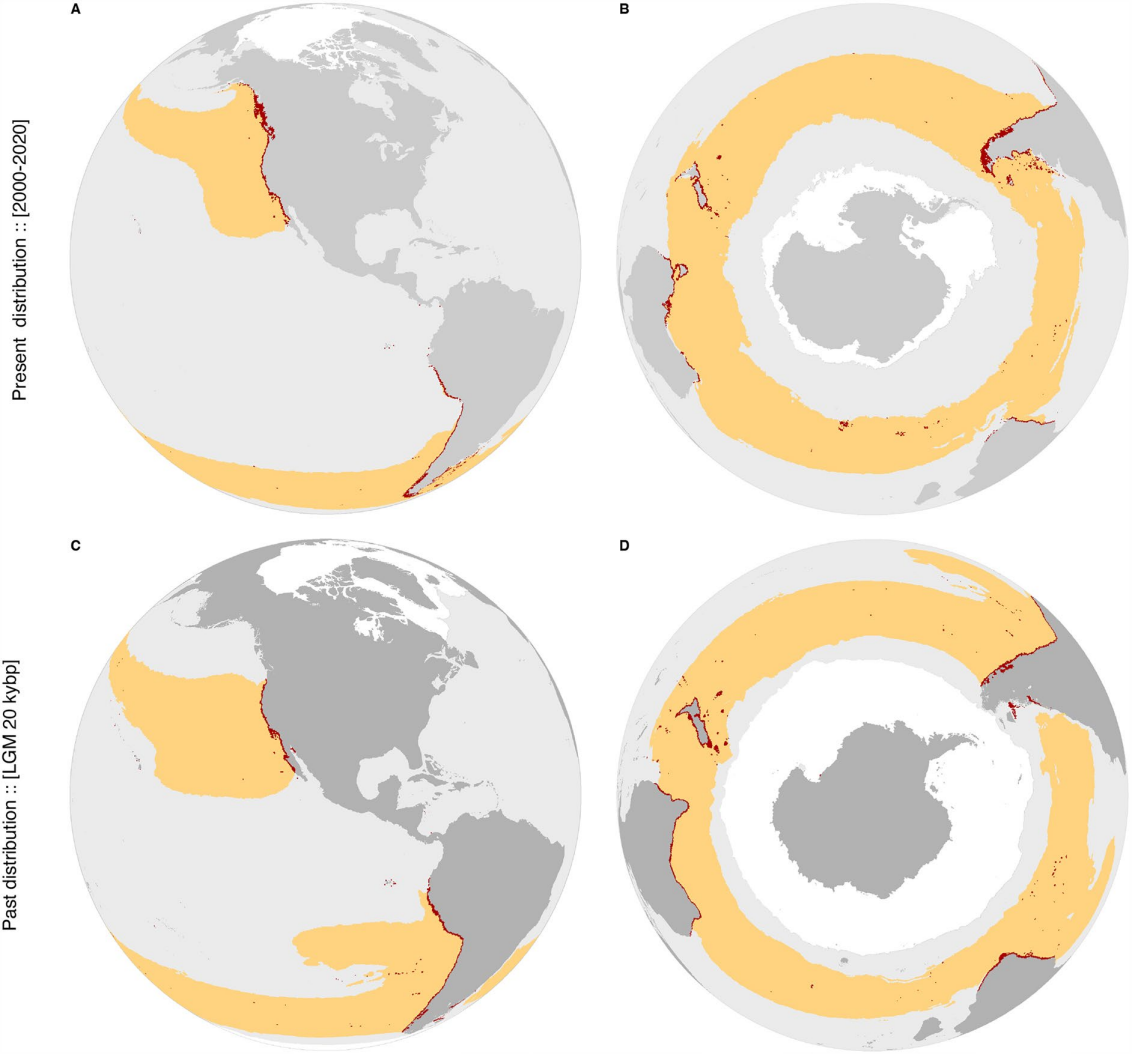
Potential distribution of *Macrocystis pyrifera* estimated with Species Distribution Modelling for (panels **A** and **B**) the present and (panels **C** and **D**) the last glacial maximum (yellow and red colors depicting surface and subtidal habitats down to 30 m depth, respectively). Sea ice extent is depicted in white. Detailed maps per continent available in Figs. 6–9 in S3. Occurrence records used in Species Distribution Modelling in Fig. 10 in S3.

The models showed good potential for transferability (CV AUC > 0.9; CV Sensitivities > 0.9; Table 2) and the predictions performed for the present largely matched the known distribution of the species, as verified by sensitivity (i.e., true positive rate) > 0.95 and AUC > 0.94 (Table 2; Fig. 1). Comparing predictions with the species’ realized distribution, as inferred by reports of occurrence records (Fig. 10 in S3), shows some degree of overprediction in Alaska (from Palma Bay to Kodiak Island, ~ 900 km coastline), Chile (from Antofagasta to Huasco, Atacama, ~ 500 km coastline), Southern Africa (from Paternoster, South Africa, to Kunene River, Namibia, ~ 1,500 km coastline). To a lower extent, overprediction was detected in Western Australia (~ 300 km coastline) and Southern Australia (~ 400 km coastline). These are regions where the species is not reported to occur, but where the models predicted suitable conditions based on the predictor variables considered. Similarly, the species was continuously predicted along the coastlines of Washington (USA) and Southern Oregon, where reports of occurrence records are elusive (Fig. 10 in S3). During the LGM, the models estimated a 40% range contraction within the regions sampled for genetic data (Table 3). During that past period, unsuitable areas might have occurred in the high latitudinal regions of Alaska to Washington state (USA), and throughout most of the Southern Ocean, Tierra del Fuego (Argentina) and Tasmania (Fig. 1; Table 3). Conversely, the low latitude range limits might have remained stable (e.g., California to Mexico, Northern Chile, Peru, Australia and New Zealand). Among the sampled regions, Channel Islands to Mexico was the only one predicted with wider suitable habitats in the LGM when compared to the present (Table 3). Results also indicated that other regions, like Southeastern Africa, Western and Eastern Australia and Northern New Zealand, might have had wider suitable habitats for *M. pyrifera* during the LGM (Figs. 6–9 in S3), with ranges potentially expanding beyond its present-day modelled distribution. Australia is in the same genetic group as Patagonia and the sub-Antarctic islands (see genetic results below), and these latter ones suffered local extinctions due to ice during the LGM, therefore the genetic group as a whole increased in area from the LGM to the present (Table 3), even though that was not the case in Australia.

**Table 2:** Performance of species distribution models inferred with cross-validation (CV) and the final predictive surfaces, both for the northern and southern hemispheres.

Model	AUC (CV)	Sensitivity (CV)	AUC (final)	Sensitivity (final)
Northern hemisphere	0.934 ± 0.045	0.902 ± 0.094	0.969	0.951
Southern hemisphere	0.907 ± 0.136	0.901 ± 0.145	0.946	0.979

**Table 3:** Potential distributional area (and rate of change) estimated with Species Distribution Modelling per geographic region, where genetic sampling took place (linked to the second level of genetic subdivision), for the present and the Last Glacial Maximum.

	Area (× 1000 km^2^)	Area change (%)	
Geographic region (genetic cluster)	LGM	Present	LGM to present
G1: Alaska, Canada & Monterey	8.66	155.83	** + 94.44**
G2: Santa Cruz to Point San Luis	13.33	13.55	+ 1.63
G3: Point Conception region	5.60	5.74	+ 2.36
G4: Channel Islands to Mexico	24.68	22.92	− 1.07
Ad-mixture [Peru]	15.69	15.86	+ 1.06
G5: N. Chile & New Zealand	626.55	758.38	+ 17.38
G6: Patagonia, Australia & Subant. Islands	289.49	667.75	** + 56.65**
All regions	981.99	1640.02	+ 40.12

Bold values depicting range shifts > 50% of area from the LGM to the present.

The models predicting suitable surface conditions (i.e., exclusively for rafting) showed disjunct potential distributions between the northern and southern hemispheres during the LGM and the present. Throughout the Southern Ocean, models showed broad suitable conditions for rafting with present-day conditions, while during the LGM, a discontinuity might have occurred along southern Chile and Tierra del Fuego (Argentina).

### Genetic structure and diversity

**Figure 2:**
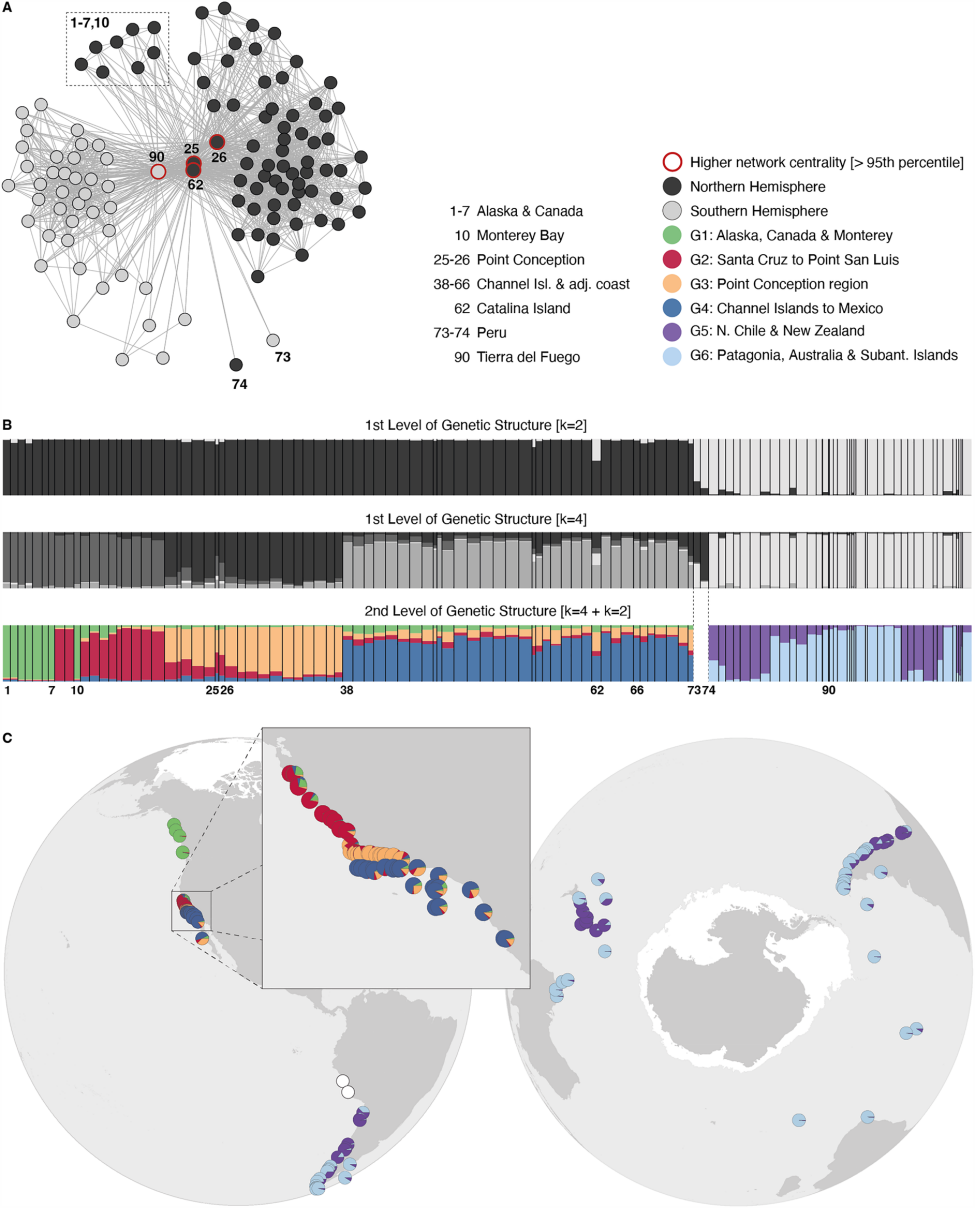
Genetic structure of *Macrocystis pyrifera*. (panel **A**) Network analysis based on shared allele distance. Red circles in the network identify sites with higher network centrality (> 95th percentile of centrality). (panel **B**) ﻿Structure analysis for the first and second hierarchical levels of subdivision with bars representing the proportion of individual assignments to each cluster. Analyses using the DeltaK criterion^68^ to infer the number of K clusters available in S3: first hierarchical level with K = 2 or 4; second hierarchical level with K = 4 and K = 2. Colors depict distinct genetic groups and the numbers label populations of interest. (panel **C**) Second hierarchical level of genetic subdivision (proportion of individual assignments to each cluster). Sea ice extent is depicted in white.

A total of 156 alleles were amplified across 3872 genotyped specimens (range of alleles per locus: 15–39). The ﻿Evanno criteria applied to the first hierarchal level of Structure revealed 2 genetic clusters associated to the northern and southern hemispheres, and 4 clusters dividing the northern hemisphere in (1) Alaska to Point San Luis, (2) Point Conception region and (3) Channel Islands to Mexico (Figs. 1, 2; S3). This level of structure showed admixture in Catalina Island, and depending on the selected number of clusters (2 or 4), assigned Peru (sites 73 and 74) to the northern hemisphere (cluster G3, Point Conception region) or to the southern hemisphere (Fig. 2). The second hierarchical level of Structure subdivided the northernmost cluster (Alaska to Point San Luis) into Alaska, Canada and Monterey, and Santa Cruz to Point San Luis. Admixture was mostly verified in Point Conception (sites 25 and 26; Fig. 2). The southern hemisphere was divided into a broad region including northern Chile and New Zealand, and into Patagonia, Australia and the Subantarctic Islands. The network analysis supported the two main clusters of the northern and southern hemispheres (Modularity p-value test: 0.001) and showed the two sites in Peru belonging to the northern (site 74) and southern clusters (site 73; Fig. 2). This analysis further showed Point Conception, Catalina Island and Tierra del Fuego (sites 25, 26, 62 and 90; Fig. 2) with higher eigenvector centrality (> 95th percentile).

**Figure 3:**
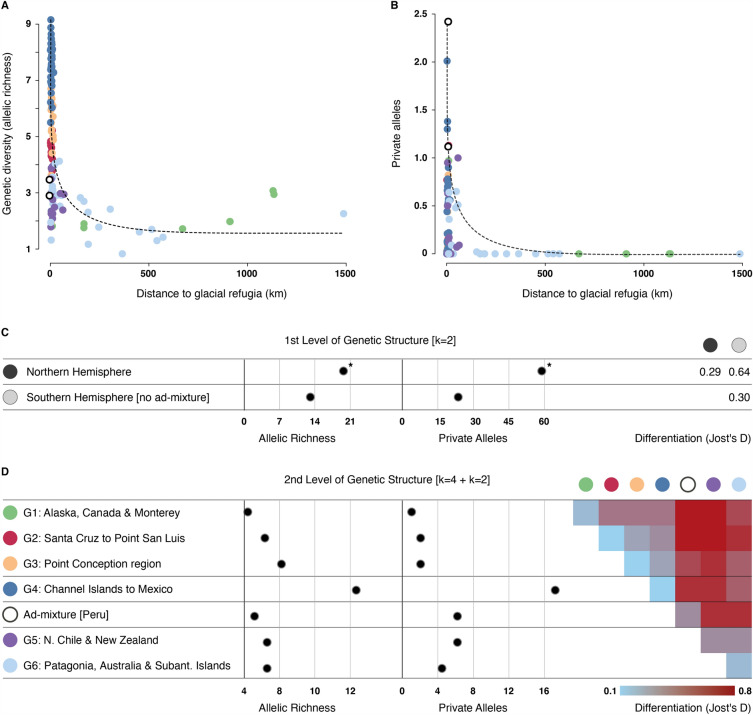
Standardized genetic diversity per site as a function of (panels **A** and **B**) distance to closest glacial refugia (version considering the natural logarithm of distance in Fig. 4 in S3). Colors depicting the assignment of populations to the different genetic clusters (as in Fig. 2). Standardized diversity per genetic group for the (panel **C**) first and (panel **D**) second hierarchical levels of genetic subdivision. Population pairwise differentiation (Jost's D, average) between genetic groups inferred for the (panel **C**) first and (panel **D**) second levels of genetic subdivision. Asterisks depict groups with significantly higher genetic diversity.

The two main clusters associated with the northern and southern hemispheres showed high differentiation (Jost’s D: 0.64; Fig. 3C). At a lower level of genetic structure, Peru (admixture region) and the cluster comprising northern Chile and New Zealand were most differentiated among themselves and from all remaining clusters (Fig. 3D). In terms of genetic diversity, results show allelic richness per site higher in the Channel Islands and San Mateo Point (USA), followed by San Diego region (USA) and Mexico (Â > 8; S1). Lower diversity values were found in the regions predicted as extinct during the LGM (i.e., Patagonia, throughout Marion, Gough and Macquarie Subantarctic Islands and in Canada and Alaska). Number of private alleles were higher in the Channel Islands (particularly in San Clemente and Santa Barbara Islands), in the two sampled sites of Peru, in Monterey (USA) and New Zealand (PÂ > 1; S1). More than 50 sites distributed throughout the sampled regions exhibited no private alleles. Overall, allelic richness outside glacial refugia was always < 3, with the exception of two sites in Alaska (USA), and the average number of private alleles was < 1 at distances > 55 km from refugia and = 0 at distances > 150 km from refugia (Fig. 3A, B). The sites Sitka (Alaska), Craig (Alaska) and Bamfield Island (Canada) of the northern hemisphere and Grytviken (Subantarctic Island) and Murray Channel (Tierra del Fuego) of the southern hemisphere distanced > 500 km from the closest glacial refugia (Table S1). Considering the first level of genetic clustering, results show diversity (allelic richness and number of private alleles) significantly higher in the northern hemisphere (Fig. 3C). The second level of structure revealed higher diversity in the cluster from Channel Islands to Mexico and lower diversify in that of Alaska and Canada. In the southern hemisphere the number of private alleles were higher in Peru and in the northern Chile and New Zealand clusters.

## Discussion

﻿The rare opportunity to study a biological model with replication of postglacial expansions in both northern and southern hemispheres allowed us to demonstrate that the distribution of intraspecific marine biodiversity can be structured by comparable evolutionary forces across the global ocean. By combining independent theoretical distribution modelling with empirical genetic data, we show how past range shifts (c. 20 Kyr ago to the present) shaped refugial regions in both hemispheres, where *Macrocystis pyrifera* retained higher and unique genetic diversity, and how significant post-glacial expansions left ~ 40% of the sampled distribution with homogenous reduced diversity, regardless the distance to refugia. Genetic patterns further show the northern hemisphere with significantly higher genetic diversity and number of private alleles, supporting previous hypotheses^46^ of an ancestral origin of the species in this region. In particular, the genetic hotspots found at the Channel Islands (California; putative global origin of *M. pyrifera*) and in Peru (highest number of private alleles), potentially involved in the ancestral radiation of lineages, are suggested here as priority conservation areas under Climate Change Integrated Conservation Strategies for potentially endangered phylogeographic lineages^72^. The distribution of 6 distinct clusters, with strong genetic discontinuities corresponding to previously described phylogeographic breaks of additional species and well-known oceanographic barriers shaped by ocean currents and habitat discontinuities, allow making broad generalizations about the process driving marine phylogeography, particularly important for ecosystems structuring species.

### Climate changes structuring genetic diversity across the hemispheres

﻿The SDM approach showed the potential distribution of *Macrocystis pyrifera* (i.e., climatic suitable habitats) mainly driven by thermal conditions (i.e., minimum and maximum extremes), which is typical for marine forest species modelled at the scales of our study^2,7^. The estimated thermal tolerance of the species between approx. 2 and 24 °C largely agreed with empirical physiological studies^73,74^, which together with the evidence found of niche conservatism between hemispheres (i.e., identical ecological niches; S2) provided strong support for the models, particularly important when performing temporal transferability to estimate demographic changes^57,75^. The high performance shown by our SDM approach is expected when using machine learning algorithms with proper hyper-parameterization and monotonicity constrains^49^, fitting biologically meaningful predictors against comprehensive datasets of distribution records^7,76^. Despite the general agreement found between predicted suitable habitats and reported occurrence records (i.e., high sensitivity rate), overprediction was found in Alaska, Chile, and Southwestern Africa, and, to a lower extent, in Western and Southern Australia^52^, and along the coastlines of Washington and Oregon states (USA). While the models predicted favorable conditions, additional predictor variables not considered might be preventing its occurrence. Specifically, the lack of rocky substrate^43^, interspecific competition^77^, limited light conditions^78^, and restricted dispersal potential^29^ can be excluding the species from such areas predicted as suitable.

Hindcasting the models to LGM climatologies estimated a 40% range contraction along the regions sampled for genetics. This was mostly evident from Alaska to Vancouver Island and throughout most of sub-Antarctic Islands, Southern Patagonia and Tasmania. Conversely, lower latitude ranges (e.g., California to Mexico, Northern Chile, Peru, Australia and New Zealand) might have provided suitable habitats from the LGM to the present. The rate and magnitude of these changes are not uncommon for cold-temperate species^6,9–11,14^, but were never addressed before with the same biological model exposed to the LGM conditions of both hemispheres in a SDM framework. This allowed identifying multiple mid- to low latitude refugia at global scales, for which peri-glacial margins largely match additional genetic studies; for instance, Vancouver Island adjacent to the Laurentide Ice sheet in the northern hemisphere^79^ and Patagonia and the Falkland Islands in the southern hemisphere^24^. As anticipated, populations within refugia showed higher and unique genetic diversity levels, as long-term persistence rendered the possibility of population to accumulate and retain regional diversity levels^8,9,14,80^. Outside refugia, where range shifts might have occurred, diversity levels were always lower regardless the hemisphere, with private alleles reduced to negligible values < 1 at distances > 55 km of refugia, and to 0 at distances > 150 km. This skewed pattern of diversity occurring at such short distances of closest refugia (but not from the putative origin region; Fig. 5 in S3) likely resulted from consecutive founder effects occurring during post-glacial range expansions, which have left homogeneous landscapes with lower diversity levels in the two hemispheres^81^. This pattern might have been maintained by density barrier effects, halting the expansion of distinct immigrant genes^82,83^.

Results also highlighted the role of suitable habitat changes (i.e., predicted areas) as a proxy of effective population size changes through time^11^. In particular, the genetic hotspot found from Channel Islands to Mexico, with striking higher diversity compared to anywhere else, was the only sampled region that increased suitable habitats during the LGM. An additional study shows this region with even larger habitats (up to threefold) from the LGM to the mid-Holocene^42^. ﻿Large and stable habitats can potentially maintain population sizes large enough to counteract the genetic effects of inbreeding depression and drift over time^84^. This contrasts with regions like Patagonia and sub-Antarctic islands that might have experienced severe population reductions, while not complete extinct (up to 56% LGM contraction), and today show reduced diversity. But comparing diversity levels between sites / genetic groups must consider additional drivers beyond potential habitat changes through time. For instance, the major genetic bottleneck during the colonization of the southern hemisphere^46^ (see “Discussion” below on “Global phylogeographic patterns”) may have strongly reduced diversity of new colonized areas, despite the subsequent relative size of persisting refugia. Accordingly, outside the putative region of *Macrocystis* origin, even populations within estimated regionally persistent areas, may presently contain lower diversity than expected in the ancient locations of its ancestral evolutionary history.

Genetic erosion within refugia can further occur due to the exposure of individuals to peripheral niche conditions at low latitude range margins, typically characterized by nutrient-deprived warmer waters^30,85^. In the present study, Mexico (northern hemisphere) and Peru (southern hemisphere) can represent such cases. Despite being predicted within refugia, considering their high private diversity levels and SDM estimates, both regions show low allelic richness. There, studies following demographic changes of giant kelp have recurrently linked the extreme conditions of El Niño events to local population extinctions^32,86^, which might have produced bottlenecks and shifted genetic baselines^34^. Cryptic persistence in deep colder waters or microscopic gametophyte stages, as suggested elsewhere^87,88^, could have counteracted complete genetic losses and safeguarded the observed unique diversity. In this context, climate change scenarios are projected to shift the 24 °C isotherm matching the species’ thermal tolerance to more poleward regions, hundreds of km beyond these sites, particularly in the no mitigation scenarios overlooking the Paris Agreement initiative^89^. The complete loss of unique genes, as recently reported for the *Ecklonia* forests of Oman^90^, is of particular concern^30^, calling for considering both warm range edges of *M. pyrifera* as priority for conservation in light of Climate Change Integrated Conservation Strategies for potentially endangered phylogeographic lineages^72^.

### Global phylogeographic patterns

Results showed *M. pyrifera* composed by two main genetic clusters, which coincide with the division of both hemispheres, followed by a second and more complex level of structure comprising six clusters. All clusters showed significant pairwise differentiation levels, largely suggesting the effect of genetic drift, not compensated by regular gene flow. Compared to the southern hemisphere, the northern hemisphere showed significantly higher genetic diversity and up to fourfold more private alleles (i.e., genetic diversity endemic of the northern hemisphere), rendering it the potential ancient origin of the species. This is supported by previous studies also hypothesizing the southern hemisphere distribution of *M. pyrifera* and additional cold-temperate species of macroalgae resulting from northward introductions^46,91^. The centrality patterns found in both genetic structure and network analyses point to Catalina Island bridging the hemispheres through the region sampled in Peru, a hypothesis raised elsewhere^46^. This process can explain the admixture levels found in Peru, and may have occurred by stepping-stone through unsampled tropical regions where the species occurred, or still occurs in elusive habitats (e.g., Socorro Island in Mexico)^92^, yet in a distant past beyond the LGM, as SDM does not suggest the possibility of rafting between the hemispheres. Additional temperature reconstructions for this tropical area match the sea surface temperature anomaly of our data during the LGM of approx. − 2 °C, reinforcing a plausible long-term barrier for rafting. Earlier periods like the pre- or mid-Pleistocene climate transition (620–435 Kyr ago and 870–620 Kyr ago) could have provided suitable conditions for the stepping-stone process, as suggested for the red algae *Callophyllis variegata*^91^.

Marked genetic structure hinders the inference of other colonization pathways, with the exception for the regions more recently colonized (i.e., Alaska, Canada and most sub-Antarctic Islands). Structure and network analyses (degree centrality) suggest Alaska and Canada potentially originating from Monterey Bay, and the sub-Antarctic Islands being colonized by Tierra del Fuego or a neighboring persistent location (e.g., Falkland Islands), where the species could have dispersed^9,24,93^; this contrasts with additional persistent areas (e.g., southern Chile) from which dispersal might have been hindered by the major discontinuity for rafting occurring along southern Chile and Tierra del Fuego, as suggested by the SDM*.*

The genetic divisions observed in both hemispheres are corroborated by previous studies of *M. pyrifera*^38,39,46^, and reinforce the role of oceanographic currents and habitat discontinuity shaping the genetic structure of the species, as previously studied along the Californian coastline^43,44^. In the northern hemisphere, the deflection of the Alaska and California currents^94^ can explain the division between G1 (Alaska, Canada & Monterey) and G2 (Santa Cruz to Point San Luis), while the well-known biogeographic barrier of Point Conception^95^ defines a genetic cluster per se. In the southern hemisphere, the split of the Antarctic Circumpolar Current into the northward Humboldt current and the southward Cape Horn current shapes a barrier at − 42° latitude matching the observed division between Patagonia and N. Chile (G5 and G6). The genetic discontinuities in this region were also associated with founder effects during post-glacial colonization of Patagonia for other macroalgae species, as well as for invertebrates and vertebrates^96–99^. The subdivision of Peru can result from habitat discontinuity shaped by extensive sandy beaches, or by the highly advective Humboldt Current System, which promotes off-shore transport, therefore generating a connectivity barrier to coastal populations. Lastly, the Tasman Sea, identified as a major biogeographical barrier for marine biodiversity^100^, explains the additional separation of G5 and G6, from Australia to New Zealand.

Overall, the present study with replication along the northern and southern hemisphere, highlights the role of past climate changes at global scales in structuring refugial regions where populations may display higher and unique genetic diversity, compared to more recent populations resulting from post-glacial expansions. Additional drivers such as regional population size changes, genetic bottlenecks produced by major phylogeographic events (e.g., the colonization of the southern hemisphere) and the exposure to peripheral niche conditions in warm range edges might have further been key to structuring global diversity levels. The study also highlights the role of major oceanographic currents and habitat discontinuities, coupled with the density barrier effects of previously established populations, restricting the homogenization of distinct genetic lineages over time, as suggested for other kelp species^3,98^. In contrast, long-distance dispersal by rafting must be a rare process^29^, but crucial to the colonization of unoccupied suitable habitats following glacial events. Together, these processes shaped disjunct genetic lineages at short distances (scales of tens to hundreds of km), rendering them of higher conservation value in the face of future climate changes, particularly if one considers the potential loss of unique alleles at low latitude range margins, and the multiple ecological, and socioeconomic services provided by giant kelp forests^27^. The insights provided with replication across hemispheres, contribute to a better understanding of the genetic effects of past climate changes, and may be generalizable to additional cold-temperate species, particularly for other important ecosystem-structuring species.

## Supplementary Information

Supplementary Table 1.

Supplementary Information.

Supplementary Figures.

## Data Availability

Genetic data, occurrence records and climate layers for species distribution modeling are openly available at https://doi.org/10.6084/m9.figshare.20390715

## References

[1] Provan J (2013). The effects of past, present and future climate change on range-wide genetic diversity in northern North Atlantic marine species. Front. Biogeogr..

[2] Song X-H (2021). Climate-induced range shifts shaped the present and threaten the future genetic variability of a marine brown alga in the Northwest Pacific. Evol. Appl..

[3] Assis J (2018). Past climate changes and strong oceanographic barriers structured low-latitude genetic relics for the golden kelp Laminaria ochroleuca. J. Biogeogr..

[4] Clark PU, Mix AC (2002). Ice sheets and sea level of the last glacial maximum. Quat. Sci. Rev..

[5] Peltier WR (1994). Ice age paleotopography. Science.

[6] Neiva J (2018). Glacial vicariance drives incipient speciation in the amphi-boreal kelp Saccharina latissima. Sci. Rep..

[7] Assis J, Araújo MB, Serrão EA (2017). Projected climate changes threaten ancient refugia of kelp forests in the North Atlantic. Glob. Chang. Biol..

[8] Assis J (2016). Deep reefs are climatic refugia for genetic diversity of marine forests. J. Biogeogr..

[9] Lau SCY, Wilson NG, Silva CNS, Strugnell JM (2020). Detecting glacial refugia in the Southern Ocean. Ecography.

[10] Neiva J, Assis J, Fernandes F, Pearson GA, Serrão EA (2014). Species distribution models and mitochondrial DNA phylogeography suggest an extensive biogeographical shift in the high-intertidal seaweed Pelvetia canaliculata. J. Biogeogr..

[11] Assis J, Serrão EA, Claro B, Perrin C, Pearson GA (2014). Climate-driven range shifts explain the distribution of extant gene pools and predict future loss of unique lineages in a marine brown alga. Mol. Ecol..

[12] Hewitt GM (1999). Post-glacial re-colonization of European biota. Biol. J. Lin. Soc..

[13] Kaufman D (2020). Holocene global mean surface temperature, a multi-method reconstruction approach. Sci. Data.

[14] Maggs CA (2008). Evaluating signatures of glacial refugia for north atlantic benthic marine taxa. Ecology.

[15] Neiva J, Pearson GA, Valero M, Serrão EA (2010). Surfing the wave on a borrowed board: Range expansion and spread of introgressed organellar genomes in the seaweed *Fucus ceranoides* L.. Mol. Ecol..

[16] Castilho R, Grant WS, Almada VM (2013). Biogeography and phylogeography of the Atlantic. Front. Biogeogr..

[17] Hewitt GM (2004). Genetic consequences of climatic oscillations in the quaternary. Philos. Trans. R. Soc. Lond. B Biol. Sci..

[18] Hughes PD, Gibbard PL, Ehlers J (2020). The missing glaciations of the middle pleistocene. Quat. Res. (United States).

[19] Benz V, Esper O, Gersonde R, Lamy F, Tiedemann R (2016). Last glacial maximum sea surface temperature and sea-ice extent in the Pacific sector of the Southern Ocean. Quat. Sci. Rev..

[20] Guillemin ML, Valero M, Faugeron S, Nelson W, Destombe C (2014). Tracing the trans-pacific evolutionary history of a domesticated seaweed (Gracilaria chilensis) with archaeological and genetic data. PLoS ONE.

[21] Fraser CI, Nikula R, Waters JM (2011). Oceanic rafting by a coastal community. Proc. Biol. Sci. R. Soc..

[22] Fraser CI, Spencer HG, Waters JM (2009). Glacial oceanographic contrasts explain phylogeography of Australian bull kelp. Mol. Ecol..

[23] Guillemin ML, Dubrasquet H, Reyes J, Valero M (2018). Comparative phylogeography of six red algae along the Antarctic Peninsula: Extreme genetic depletion linked to historical bottlenecks and recent expansion. Polar Biol..

[24] Fraser CI, Nikula R, Ruzzante DE, Waters JM (2012). Poleward bound: Biological impacts of Southern Hemisphere glaciation. Trends Ecol. Evol..

[25] Peterson AT (2011). Ecological niche conservatism: A time-structured review of evidence. J. Biogeogr..

[26] Wiens JJ (2010). Niche conservatism as an emerging principle in ecology and conservation biology. Ecol. Lett..

[27] Graham MH, Vásquez JA, Buschmann AH (2007). Global ecology of the giant kelp. Oceanogr. Mar. Biol..

[28] Reed DC, Schroeter SC, Raimondi PT (2004). Spore supply and habitat availability as sources of recruitment limitation in the giant kelp *Macrocystis pyrifera* (Phaeophyceae). J. Phycol..

[29] Batista MB (2018). Kelps’ long-distance dispersal: Role of ecological/oceanographic processes and implications to marine forest conservation. Diversity (Basel).

[30] Hampe A, Petit RJ (2005). Conserving biodiversity under climate change: The rear edge matters. Ecol. Lett..

[31] Provan J, Maggs CA (2012). Unique genetic variation at a species’ rear edge is under threat from global climate change. Proc. R. Soc. B Biol. Sci..

[32] Cavanaugh KC, Reed DC, Bell TW, Castorani MCN, Beas-Luna R (2019). Spatial variability in the resistance and resilience of giant kelp in southern and Baja California to a multiyear heatwave. Front. Mar. Sci..

[33] Butler CL, Lucieer VL, Wotherspoon SJ, Johnson CR (2020). Multi-decadal decline in cover of giant kelp *Macrocystis pyrifera* at the southern limit of its Australian range. Mar. Ecol. Prog. Ser..

[34] Assis J (2013). High and distinct range-edge genetic diversity despite local bottlenecks. PLoS ONE.

[35] Steneck RS (2002). Kelp forest ecosystems: Biodiversity, stability, resilience and future. Environ. Conserv..

[36] Filbee-Dexter K (2020). Ocean forests hold unique solutions to our current environmental crisis. One Earth.

[37] Filbee-Dexter K, Wernberg T (2020). Substantial blue carbon in overlooked Australian kelp forests. Sci. Rep..

[38] Camus C, Faugeron S, Buschmann AH (2018). Assessment of genetic and phenotypic diversity of the giant kelp, *Macrocystis pyrifera*, to support breeding programs. Algal Res..

[39] Johansson ML (2015). Seascape drivers of *Macrocystis pyrifera* population genetic structure in the northeast Pacific. Mol. Ecol..

[40] MacAya EC, Zuccarello GC (2010). Genetic structure of the giant kelp *Macrocystis pyrifera* along the southeastern Pacific. Mar. Ecol. Prog. Ser..

[41] Salavarría E, Macaya E, Gil-Kodaka P, Paul S, Troccoli L (2018). Haplotype diversity of *Macrocystis pyrifera* (Phaeophyceae: Laminariales) in the central and southern coast of Peru. Panam J. Aquat. Sci..

[42] Graham MH, Kinlan BP, Grosberg RK (2010). Post-glacial redistribution and shifts in productivity of giant kelp forests. Proc. Biol. Sci. R. Soc..

[43] Alberto F (2010). Habitat continuity and geographic distance predict population genetic differentiation in giant kelp. Ecology.

[44] Alberto F (2011). Isolation by oceanographic distance explains genetic structure for *Macrocystis pyrifera* in the Santa Barbara channel. Mol. Ecol..

[45] Klingbeil WH, Montecinos GJ, Alberto F (2022). Giant kelp genetic monitoring before and after disturbance reveals stable genetic diversity in Southern California. Front. Mar. Sci..

[46] Coyer JA, Jason Smith G, Andersen RA (2001). Evolution of Macrocystis spp. (Phaeophyceae) as determined by ITS1 and ITS2 sequences. J. Phycol..

[47] Macaya EC, Zuccarello GC (2010). DNA barcoding and genetic divergence in the giant kelp Macrocystis (Laminariales). J. Phycol..

[48] Teske PR (2018). Mitochondrial DNA is unsuitable to test for isolation by distance. Sci. Rep..

[49] Hofner B, Müller J, Hothorn T (2011). Monotonicity-constrained species distribution models. Ecology.

[50] Elith, J. & Leathwick, J. Boosted regression trees for ecological modeling. *October* 1–22 (2011) doi:!!!!!!!!!!

[51] Assis J (2017). Bio-ORACLE v20: Extending marine data layers for bioclimatic modelling. Glob. Ecol. Biogeogr..

[52] Graham MH (2007). Global ecology of the giant kelp macrocystis : From ecotypes to ecosystems. Oceanogr. Mar. Biol..

[53] Assis J, Perrin C, Pearson GA (2014). Climate-driven range shifts explain the distribution of extant gene pools and predict future loss of unique lineages in a marine Climate-driven range shifts explain the distribution of extant gene pools and predict future loss of unique. Mol. Ecol..

[54] Assis J (2020). A fine-tuned global distribution dataset of marine forests. Sci. Data.

[55] Barbet-Massin M, Jiguet F, Albert CH, Thuiller W (2012). Selecting pseudo-absences for species distribution models: How, where and how many?. Methods Ecol. Evol..

[56] Boavida J, Assis J, Silva I, Serrão EA (2016). Overlooked habitat of a vulnerable gorgonian revealed in the Mediterranean and Eastern Atlantic by ecological niche modelling. Sci. Rep..

[57] Pearman PB, D’Amen M, Graham CH, Thuiller W, Zimmermann NE (2010). Within-taxon niche structure: Niche conservatism, divergence and predicted effects of climate change. Ecography.

[58] Martins MR, Assis J, Abecasis D (2021). Biologically meaningful distribution models highlight the benefits of the Paris agreement for demersal fishing targets in the North Atlantic Ocean. Glob. Ecol. Biogeogr..

[59] Allouche O, Tsoar A, Kadmon R (2006). Assessing the accuracy of species distribution models: Prevalence, kappa and the true skill statistic (TSS). J. Appl. Ecol..

[60] Gouvêa LP (2020). Golden carbon of Sargassum forests revealed as an opportunity for climate change mitigation. Sci. Total Environ..

[61] Elith J, Leathwick JR, Hastie T (2008). A working guide to boosted regression trees—Online appendices page 1. J. Anim. Ecol..

[62] Assis J, Araújo MB, Serrão EA (2018). Projected climate changes threaten ancient refugia of kelp forests in the North Atlantic. Glob. Chang. Biol..

[63] Wenger SJ, Olden JD (2012). Assessing transferability of ecological models: An underappreciated aspect of statistical validation. Methods Ecol. Evol..

[64] Elith J, Leathwick JR, Hastie T (2008). A working guide to boosted regression trees. J. Anim. Ecol..

[65] Jiménez-Valverde A, Lobo JM (2007). Threshold criteria for conversion of probability of species presence to either–or presence–absence. Acta Oecologica.

[66] Alberto F (2009). Microsatellite markers for the giant kelp *Macrocystis pyrifera*. Conserv. Genet..

[67] Pritchard JK, Stephens M, Donnelly P (2000). Inference of population structure using multilocus genotype data. Genetics.

[68] Evanno G, Regnaut S, Goudet J (2005). Detecting the number of clusters of individuals using the software STRUCTURE: A simulation study. Mol. Ecol..

[69] Rozenfeld AF (2008). Network analysis identifies weak and strong links in a metapopulation system. Proc. Natl. Acad. Sci. USA.

[70] Newman MEJ (2006). Modularity and community structure in networks. Proc. Natl. Acad. Sci..

[71] Whitlock MC (2011). GST and D do not replace FST. Mol. Ecol..

[72] Hannah L, Midgley GF, Millar D (2002). Climate change-integrated conservation strategies. Glob. Ecol. Biogeogr..

[73] Tom Dieck IT, Dieck IT (1993). Temperature tolerance and survival in darkness of kelp gametophytes (Laminariales, Phaeophyta)—Ecological and biogeographical implications. Mar. Ecol. Prog. Ser..

[74] Rothäusler E (2009). Effect of temperature and grazing on growth and reproduction of floating Macrocystis spp. (phaeophyceae) along a latitudinal gradient. J. Phycol..

[75] Hu Z-M (2021). Intraspecific genetic variation matters when predicting seagrass distribution under climate change. Mol. Ecol..

[76] Fragkopoulou E, Serrão EA, Horta PA, Koerich G, Assis J (2021). Bottom trawling threatens future climate refugia of rhodoliths globally. Front. Mar. Sci..

[77] Mpakairi KS (2017). Missing in action: Species competition is a neglected predictor variable in species distribution modelling. PLoS ONE.

[78] Krause-jensen D (2020). Imprint of climate change on Pan-Arctic marine vegetation. Front. Mar. Sci..

[79] Neiva J (2017). Glacial vicariance drives phylogeographic diversification in the amphi-boreal kelp Saccharina latissima. Sci. Rep..

[80] Provan J, Bennett KD (2008). Phylogeographic insights into cryptic glacial refugia. Trends Ecol. Evol..

[81] Excoffier L, Foll M, Petit RJ (2009). Genetic consequences of range expansions. Annu. Rev. Ecol. Evol. Syst..

[82] Waters JM, Fraser CI, Hewitt GM (2013). Founder takes all: Density-dependent processes structure biodiversity. Trends Ecol. Evol..

[83] Neiva J, Pearson GA, Valero M, Serrão EA (2012). Fine-scale genetic breaks driven by historical range dynamics and ongoing density-barrier effects in the estuarine seaweed *Fucus ceranoides* L.. BMC Evol. Biol..

[84] Wang J, Santiago E, Caballero A (2016). Prediction and estimation of effective population size. Heredity.

[85] Nicastro KR (2013). Shift happens: Trailing edge contraction associated with recent warming trends threatens a distinct genetic lineage in the marine macroalga Fucus vesiculosus. BMC Biol..

[86] Arafeh-Dalmau N (2019). Extreme Marine heatwaves alter kelp forest community near its equatorward distribution limit. Front. Mar. Sci..

[87] Ladah LB, Zertuche-González JA (2007). Survival of microscopic stages of a perennial kelp (*Macrocystis pyrifera*) from the center and the southern extreme of its range in the Northern Hemisphere after exposure to simulated El Niño stress. Mar. Biol..

[88] Ladah, L. B. & Zertuche-González, J. A. Giant kelp (*Macrocystis pyrifera*) survival in deep water (25–40 m) during El Niño of 1997–1998 in Baja California, Mexico. *Botanica Marina* **47**, (2004).

[89] Assis J (2017). Bio-ORACLE v2.0: Extending marine data layers for bioclimatic modelling. Glob. Ecol. Biogeogr..

[90] Coleman MA (2022). Loss of a globally unique kelp forest from Oman. Sci. Rep..

[91] Bringloe TT, Macaya EC, Saunders GW (2019). The phylogeographic history of amphitropical Callophyllis variegata (Florideophyceae, Rhodophyta) in the Pacific Ocean. Algae.

[92] Taylor, W. R. *Pacific marine algae of the Allan Hancock Expeditions to the Galapagos Islands*. (Allan Hancock Pacific Expeditions, 1945).

[93] Fraser CI, Nikula R, Spencer HG, Waters JM (2009). Kelp genes reveal effects of subantarctic sea ice during the Last Glacial Maximum. Proc. Natl. Acad. Sci. USA.

[94] Freeland HJ (2006). What proportion of the North Pacific current finds its way into the Gulf of Alaska?. Atmos. Ocean.

[95] Cassone BJ, Boulding EG (2006). Genetic structure and phylogeography of the lined shore crab, Pachygrapsus crassipes, along the northeastern and western Pacific coasts. Mar. Biol..

[96] Guillemin ML (2016). Phylogeography of seaweeds in the south east pacific: Complex evolutionary processes along a latitudinal gradient. Seaweed Phylogeogr..

[97] Haye PA (2014). Phylogeographic structure in benthic marine invertebrates of the southeast pacific coast of Chile with differing dispersal potential. PLoS ONE.

[98] Fraser CI, Thiel M, Spencer HG, Waters JM (2010). Contemporary habitat discontinuity and historic glacial ice drive genetic divergence in Chilean kelp. BMC Evol. Biol..

[99] Tellier F, Meynard AP, Correa JA, Faugeron S, Valero M (2009). Phylogeographic analyses of the 30°s south-east Pacific biogeographic transition zone establish the occurrence of a sharp genetic discontinuity in the kelp Lessonia nigrescens: Vicariance or parapatry?. Mol. Phylogenet. Evol..

[100] Costello MJ (2017). Marine biogeographic realms and species endemicity. Nat. Commun..

