# Supplementary material for: Past climate-driven range shifts structuring intraspecific biodiversity levels of the giant kelp (Macrocystis pyrifera) at global scales
Source: Sci Rep. 2023 Jul 25;13:12046. doi: 10.1038/s41598-023-38944-7 (PMC10368654; doi:10.1038/s41598-023-38944-7)
Supplement: Supplementary file 1 — Supplementary Table 1. [file 41598_2023_38944_MOESM1_ESM.docx]

**Supplementary information 1**

**Genetic diversity levels of *Macrocystis pyrifera***

Table 1. List of samples of *Macrocystis pyrifera*. Site code (#), site name, country / main region, longitude (Lon) and latitude (Lat) in decimal degrees, sample size (n), standardized allelic richness (Â), standardized number of private alleles (PÂ) and expected heterozygosity (*H*e) for a common sample size of 20 individuals (resampling was allowed for smaller sample sizes), as well as distance to glacial refugia, as predicted with Species Distribution Modelling. Bold for Â values above 7.5 and PÂ values above 1.

| Code | Site | Country / Region | Lon | Lat | n | A | PA | He | Dist (km) |
| --- | --- | --- | --- | --- | --- | --- | --- | --- | --- |
| 1 | Sitka | USA | -135.415 | 57.061 | 30 | 3.06±0.12 |  | 0.4±0.02 | 1133.85 |
| 2 | Sitka | USA | -135.348 | 57.042 | 30 | 3.19±0.13 |  | 0.27±0 | 1129.40 |
| 3 | Craig | USA | -133.157 | 55.475 | 29 | 1.98±0.05 |  | 0.4±0.01 | 910.34 |
| 4 | Craig | USA | -133.191 | 55.470 | 29 | 1.98±0.05 |  | 0.23±0.02 | 911.46 |
| 5 | Bamfield Island | Canada | -130.308 | 54.069 | 39 | 1.72±0.1 |  | 0.27±0.02 | 670.65 |
| 6 | Kyuquot | Canada | -126.533 | 49.600 | 25 | 1.76±0.09 |  | 0.28±0.02 | 170.31 |
| 7 | Nuchatlitz | Canada | -126.538 | 49.616 | 25 | 1.9±0.11 |  | 0.32±0.01 | 170.88 |
| 8 | Sandhill Bluff | USA | -122.152 | 36.974 | 40 | 4.2±0.22 |  | 0.32±0.01 | 11.27 |
| 9 | Terrace Point | USA | -122.063 | 36.946 | 37 | 3.78±0.21 |  | 0.36±0.01 | 9.61 |
| 10 | Monterey bay | USA | -122.034 | 36.945 | 26 | 1.82±0.05 | 0.97±0.17 | 0.27±0.03 | 10.29 |
| 11 | Monterey bay | USA | -122.034 | 36.945 | 36 | 5.23±0.29 | **1.13±0.70** | 0.34±0.02 | 10.29 |
| 12 | Asilomar | USA | -121.947 | 36.632 | 40 | 4.78±0.23 | 0.77±0.42 | 0.4±0.01 | 1.32 |
| 13 | Stillwater Cove | USA | -121.940 | 36.554 | 37 | 4.84±0.27 | 0.03±0.18 | 0.61±0.02 | 2.11 |
| 14 | Esalen | USA | -121.653 | 36.130 | 32 | 4.31±0.15 |  | 0.23±0.02 | 5.02 |
| 15 | La Cruz | USA | -121.327 | 35.714 | 19 | 4.45±0.16 |  | 0.08±0.02 | 4.41 |
| 16 | Cayucos | USA | -121.122 | 35.569 | 40 | 4.73±0.24 |  | 0.38±0.03 | 3.08 |
| 17 | Estero | USA | -121.027 | 35.474 | 39 | 4.69±0.2 | 0.03±0.18 | 0.27±0.03 | 2.22 |
| 18 | Montana de Oro | USA | -120.891 | 35.233 | 41 | 4.61±0.27 | 0.15±0.36 | 0.37±0.05 | 6.74 |
| 19 | Avila | USA | -120.713 | 35.167 | 52 | 4.33±0.3 | 0.04±0.19 | 0.21±0.03 | 4.71 |
| 20 | Purisima Point | USA | -120.626 | 34.730 | 50 | 4.89±0.29 |  | 0.62±0.02 | 15.35 |
| 21 | Arguello | USA | -120.609 | 34.547 | 16 | 4.42±0.36 |  | 0.58±0.02 | 9.15 |
| 22 | Cojo | USA | -120.501 | 34.507 | 43 | 5.26±0.37 |  | 0.57±0.01 | 6.60 |
| 23 | Jalama - Pt. Conception | USA | -120.486 | 34.474 | 49 | 5.2±0.32 |  | 0.58±0.02 | 5.39 |
| 24 | Cojo - Pt. Conception | USA | -120.422 | 34.443 | 44 | 6.36±0.34 | 0.82±0.63 | 0.57±0.02 | 8.26 |
| 25 | Bulito - Pt. Conception | USA | -120.339 | 34.457 | 15 | 1.93±0.1 |  | 0.58±0.02 | 4.38 |
| 26 | Bulito - Pt. Conception | USA | -120.339 | 34.457 | 24 | 3.62±0.2 |  | 0.57±0.02 | 4.38 |
| 27 | Bulito - Pt. Conception | USA | -120.333 | 34.459 | 51 | 5.96±0.32 | 0.04±0.2 | 0.55±0.02 | 3.84 |
| 28 | Arroyo Hondo | USA | -120.144 | 34.472 | 31 | 6.1±0.23 |  | 0.4±0.02 | 6.06 |
| 29 | Arroyo Quemado | USA | -120.121 | 34.469 | 50 | 6.17±0.35 |  | 0.53±0.03 | 8.07 |
| 30 | Naples Reef | USA | -119.953 | 34.422 | 48 | 6.31±0.33 | 0.44±0.5 | 0.53±0.03 | 5.27 |
| 31 | Isla Vista | USA | -119.858 | 34.403 | 50 | 6.71±0.41 |  | 0.61±0.02 | 3.47 |
| 32 | Goleta Bay | USA | -119.822 | 34.414 | 46 | 6.29±0.43 | 0.07±0.25 | 0.57±0.02 | 5.15 |
| 33 | Arroyo Burro | USA | -119.744 | 34.400 | 36 | 5.5±0.25 |  | 0.63±0.02 | 5.16 |
| 34 | Mohawk | USA | -119.730 | 34.394 | 44 | 6.41±0.32 |  | 0.8±0.01 | 6.06 |
| 35 | Carpinteria | USA | -119.544 | 34.392 | 50 | 6.1±0.32 | 0.02±0.15 | 0.73±0.01 | 13.44 |
| 36 | Emma Wood | USA | -119.342 | 34.291 | 33 | 5.72±0.24 |  | 0.64±0.02 | 14.05 |
| 37 | Emma Wood | USA | -119.340 | 34.293 | 32 | 5.03±0.25 |  | 0.63±0.02 | 14.40 |
| 38 | San Miguel Island | USA | -120.442 | 34.039 | 41 | 6.23±0.36 | 0.02±0.15 | 0.66±0.02 | 1.54 |
| 39 | San Miguel Island | USA | -120.399 | 34.028 | 45 | 6.87±0.38 | 0.51±0.63 | 0.2±0.01 | 5.66 |
| 40 | San Miguel Island | USA | -120.325 | 34.044 | 38 | 6.04±0.32 | 0.57±0.5 | 0.67±0.02 | 9.48 |
| 41 | San Miguel Island | USA | -120.334 | 34.018 | 47 | 6.99±0.42 | 0.05±0.21 | 0.67±0.02 | 7.62 |
| 42 | San Miguel Island | USA | -120.188 | 34.010 | 49 | **8.12±0.46** | 0.22±0.42 | 0.66±0.02 | 11.14 |
| 43 | San Miguel Island | USA | -120.212 | 33.963 | 48 | **8.37±0.41** | 0.71±0.88 | 0.64±0.01 | 7.39 |
| 44 | San Miguel Island | USA | -120.038 | 33.994 | 49 | **8.29±0.44** | 0.54±0.63 | 0.69±0.02 | 8.31 |
| 45 | San Miguel Island | USA | -120.020 | 33.931 | 45 | 7.42±0.44 | 0.03±0.17 | 0.63±0.02 | 6.50 |
| 46 | Santa Cruz island | USA | -119.737 | 34.055 | 15 | 6.82±0.45 |  | 0.62±0.02 | 8.24 |
| 47 | Santa Cruz island | USA | -119.737 | 34.055 | 20 | **7.50±0** | 0.02±0.14 | 0.6±0.02 | 8.24 |
| 48 | Santa Cruz island | USA | -119.755 | 34.057 | 48 | **8.06±0.36** | 0.05±0.21 | 0.62±0.02 | 8.29 |
| 49 | Santa Cruz island | USA | -119.546 | 34.005 | 53 | **8.52±0.39** | 0.11±0.32 | 0.7±0.02 | 4.12 |
| 50 | Santa Cruz island | USA | -119.547 | 34.046 | 52 | **8.09±0.4** | 0±0.06 | 0.2±0.01 | 0.71 |
| 51 | Anacapa Island | USA | -119.420 | 34.004 | 52 | **9.17±0.42** | 0.65±0.67 | 0.63±0.03 | 5.46 |
| 52 | Anacapa Island | USA | -119.420 | 34.015 | 49 | **8.66±0.39** |  | 0.69±0.02 | 4.63 |
| 53 | Anacapa Island | USA | -119.371 | 34.016 | 53 | **8.89±0.41** | 0.13±0.33 | 0.71±0.02 | 2.92 |
| 54 | Anacapa Island | USA | -119.361 | 34.014 | 53 | **8.32±0.4** | 0.12±0.32 | 0.72±0.02 | 3.36 |
| 55 | Leo Carrilo | USA | -118.941 | 34.042 | 15 | 5.51±0.43 |  | 0.73±0.02 | 1.64 |
| 56 | Leo Carillo | USA | -118.935 | 34.043 | 27 | 6.96±0.32 | 0.44±0.5 | 0.72±0.02 | 2.21 |
| 57 | Palos Verdes | USA | -118.396 | 33.804 | 32 | **7.6±0.35** | 0.75±0.62 | 0.7±0.02 | 5.91 |
| 58 | Santa Barbara Island | USA | -119.029 | 33.489 | 43 | **7.45±0.39** | **1.3±0.69** | 0.7±0 | 3.64 |
| 59 | Catalina Island | USA | -118.530 | 33.429 | 40 | **7.79±0.31** | 0.21±0.45 | 0.73±0.02 | 3.38 |
| 60 | Catalina Island | USA | -118.472 | 33.442 | 42 | **7.39±0.29** | 0.01±0.09 | 0.76±0.02 | 2.21 |
| 61 | Catalina Island | USA | -118.411 | 33.423 | 40 | **7.54±0.34** | 0.05±0.23 | 0.21±0.01 | 5.19 |
| 62 | Catalina Island | USA | -118.397 | 33.420 | 37 | 2.35±0.2 | 0.7±0.67 | 0.72±0.02 | 4.74 |
| 63 | Catalina Island | USA | -118.488 | 33.334 | 40 | **8.49±0.42** | 0.78±0.7 | 0.76±0.02 | 5.46 |
| 64 | San Mateo | USA | -117.590 | 33.368 | 40 | **8.14±0.41** | 0.66±0.61 | 0.75±0.02 | 3.34 |
| 65 | San Clemente Island | USA | -118.473 | 32.834 | 50 | 6.56±0.37 | **1.38±0.59** | 0.76±0.02 | 4.69 |
| 66 | San Clemente Island | USA | -118.389 | 32.857 | 26 | 7.13±0.3 | **2.01±0.98** | 0.73±0.02 | 2.41 |
| 67 | San Diego | USA | -117.255 | 32.697 | 31 | **7.88±0.27** |  | 0.67±0.04 | 8.66 |
| 68 | Punta San Miguel | USA | -117.262 | 32.682 | 28 | **7.90±0.23** |  | 0.74±0.01 | 6.90 |
| 69 | Sauzal | Mexico | -116.745 | 31.900 | 46 | **7.46±0.34** | 0.51±0.56 | 0.75±0.01 | 5.24 |
| 70 | Ensenada | Mexico | -116.696 | 31.883 | 48 | **7.78±0.38** | 0.9±0.66 | 0.69±0.02 | 9.09 |
| 71 | Punta Banda | Mexico | -115.055 | 27.862 | 39 | **7.29±0.34** | 0.56±0.64 | 0.72±0.02 | 17.04 |
| 72 | Punta Eugenia | Mexico | -115.080 | 27.851 | 21 | 3.97±0.06 | 0.01±0.1 | 0.12±0.01 | 16.21 |
| 73 | La Mina | Peru | -76.299 | -13.927 | 28 | 3.5±0.17 | **1.14±0.34** | 0.71±0.02 | 15.10 |
| 74 | Atico | Peru | -73.614 | -16.232 | 33 | 2.94±0.15 | **2.44±0.51** | 0.75±0.01 | 2.90 |
| 75 | Playa Larga | Chile | -70.096 | -21.338 | 40 | 2.26±0.18 |  | 0.81±0.01 | 12.35 |
| 76 | Caleta Constitucion | Chile | -70.591 | -23.427 | 28 | 3.88±0.19 | 0.95±0.22 | 0.76±0.01 | 4.89 |
| 77 | Antofagasta | Chile | -70.413 | -23.677 | 32 | 3.54±0.22 | 0.63±0.48 | 0.75±0.01 | 5.79 |
| 78 | Los Vilos | Chile | -71.514 | -31.908 | 26 | 2.34±0.13 |  | 0.72±0.02 | 6.16 |
| 79 | El Tabo | Chile | -71.675 | -33.454 | 40 | 1.79±0.18 |  | 0.72±0.01 | 3.10 |
| 80 | Navidad | Chile | -71.869 | -33.947 | 41 | 2.09±0.37 |  | 0.78±0.01 | 11.50 |
| 81 | Tumbes | Chile | -73.088 | -36.628 | 39 | 3.94±0.33 | 0.12±0.33 | 0.78±0.01 | 10.79 |
| 82 | Bahia Maa | Chile | -73.738 | -40.582 | 38 | 3.56±0.27 | 0.64±0.59 | 0.78±0.01 | 12.40 |
| 83 | Ancud | Chile | -73.832 | -41.866 | 40 | 4.05±0.3 | 0.09±0.29 | 0.15±0.02 | 24.74 |
| 84 | Curaco de Velez | Chile | -73.608 | -42.440 | 26 | 2.82±0.14 |  | 0.76±0.01 | 5.82 |
| 85 | Quellon | Chile | -73.615 | -43.140 | 44 | 3.11±0.26 | 0.36±0.48 | 0.75±0.01 | 12.86 |
| 86 | Aysén | Chile | -74.464 | -46.829 | 36 | 2.92±0.24 |  | 0.73±0 | 34.36 |
| 87 | Tortel | Chile | -74.478 | -48.414 | 24 | 2.92±0.1 |  | 0.51±0.01 | 4.59 |
| 88 | Natales | Chile | -72.511 | -51.735 | 2 | 1.17±0 |  | 0.42±0.02 | 192.59 |
| 89 | Magelean Strait | Tierra del Fuego | -70.854 | -53.476 | 23 | 2.42±0.1 |  | 0.33±0.05 | 303.91 |
| 90 | Timaukel | Chile | -70.154 | -53.981 | 4 | 0.83±0 |  | 0.57±0.01 | 365.11 |
| 91 | Romanche-Brozo | Tierra del Fuego | -69.483 | -54.946 | 16 | 1.61±0.1 |  | 0.53±0.05 | 451.61 |
| 92 | Murray | Tierra del Fuego | -68.381 | -54.960 | 16 | 1.7±0.17 |  | 0.27±0.02 | 514.32 |
| 93 | Falkland islands | Subant. islands | -57.752 | -51.627 | 39 | 2.91±0.2 | 0.51±0.5 | 0.24±0.06 | 96.08 |
| 94 | Puerto Lobos | Subant. islands | -65.067 | -41.997 | 10 | 1.78±0.12 |  | 0.52±0.01 | 245.76 |
| 95 | Grytviken | Subant. islands | -36.490 | -54.294 | 8 | 2.26±0.12 |  | 0.21±0.03 | 1546.37 |
| 96 | Edinburgh of the Seven Seas | Subant. islands | -12.304 | -37.063 | 9 | 1.95±0.08 |  | 0.4±0.02 | 2.60 |
| 97 | Gough Island | Subant. islands | -9.964 | -40.334 | 8 | 1.32±0.05 |  | 0.29±0.03 | 4.56 |
| 98 | Oudekraal | Subant. islands | 18.352 | -33.986 | 38 | 2.61±0.24 | 0.55±0.52 | 0.39±0.02 | 13.58 |
| 99 | Marion islands | Subant. islands | 37.834 | -46.845 | 8 | 1.3±0.07 |  | 0.26±0.02 | 241.19 |
| 100 | Warrnambool | Australia | 142.475 | -38.403 | 40 | 4.13±0.26 | 0.48±0.64 | 0.38±0.03 | 45.99 |
| 101 | Barwon Heads | Australia | 144.489 | -38.293 | 26 | 2.7±0.12 |  | 0.34±0.02 | 170.59 |
| 102 | Low Head | Tasmania | 146.799 | -41.060 | 40 | 2.83±0.18 | 0.02±0.14 | 0.31±0.01 | 152.54 |
| 103 | Howden | Australia | 147.329 | -43.008 | 29 | 2.53±0.12 | 0.65±0.48 | 0.08±0 | 49.12 |
| 104 | Fjorland South Island | New Zealand | 166.850 | -45.279 | 26 | 2.98±0.18 |  | 0.24±0.01 | 2.41 |
| 105 | Oban | New Zealand | 168.130 | -46.899 | 25 | 2.98±0.17 | 0.07±0.26 | 0.5±0.01 | 51.23 |
| 106 | Bluff | New Zealand | 168.350 | -46.599 | 38 | 2.95±0.15 | 1±0.06 | 0.4±0.01 | 69.30 |
| 107 | Nugget Point | New Zealand | 169.800 | -46.440 | 39 | 2.49±0.22 |  | 0.26±0.02 | 21.41 |
| 108 | Scarborough | New Zealand | 171.259 | -44.426 | 16 | 2.39±0.12 | 0.09±0.29 | 0.19±0.04 | 63.60 |
| 109 | Kaikoura | New Zealand | 173.684 | -42.426 | 23 | 3.49±0.15 |  | 0.36±0.02 | 4.10 |
| 110 | Wellington | New Zealand | 174.827 | -41.285 | 39 | 3.24±0.29 |  | 0.16±0.03 | 10.46 |
| 111 | Macquarie island | Subant. islands | 158.804 | -54.647 | 14 | 1.42±0.1 |  | 0.25±0.02 | 366.82 |
| 112 | Edmund island | Subant. islands | 166.255 | -50.618 | 10 | 2.76±0.16 | 0.17±0.38 | 0.26±0.02 | 12.50 |
| 113 | Campbell island | Subant. islands | 169.192 | -52.544 | 9 | 2.26±0.16 | 0.5±0.5 | 0.12±0.02 | 4.55 |
| 114 | Antipodes island | Subant. islands | 178.808 | -49.667 | 6 | 2.31±0.09 | 0.09 | 0.29±0.04 | 4.76 |
| 115 | Chathman islands | Subant. islands | -176.602 | -43.916 | 36 | 3.1±0.42 | 0.42 | 0.04±0.01 | 9.73 |
