# Supplementary material for: Past climate-driven range shifts structuring intraspecific biodiversity levels of the giant kelp (Macrocystis pyrifera) at global scales
Source: Sci Rep. 2023 Jul 25;13:12046. doi: 10.1038/s41598-023-38944-7 (PMC10368654; doi:10.1038/s41598-023-38944-7)
Supplement: Supplementary file 2 — Supplementary Information. [file 41598_2023_38944_MOESM2_ESM.docx]

**Supplementary information 2**

**Niche overlap between the two major genetic groups of *Macrocystis pyrifera***

**Methods**

Niche overlap between the northern and southern genetic groups was estimated with the probabilistic method of Swanson et al. (2015). This provides directional estimates of niche overlap under a Bayesian framework that accounts for uncertainty. The method defines the niche region (NR) of each group as the 95% probability region of the multivariate environmental space inferred with SDM. Overlap is determined as the probability of an individual from the northern hemisphere being found in the NR of southern hemisphere, and vice-versa.

An additional niche overlap analysis was performed following the hypotheses testing proposed by Warren et al. (2008). This uses two metrics of overlap, namely the Warren et al.’s’ I and the Schoener’s D, and tests for niche equivalency (or identity) by asking whether the ecological niches of both genetic hemispheres are more different than expected when drawn from the same underlying distributions. This was performed by comparing the observed D and I with a distribution of D and I generated with 10^4^ permutations randomly assigning occurrence records into one distribution or the other. A similarity test was also performed asking whether both niches are more similar to one another than expected by chance. This compared the D and I values to a null distribution of 10^4^ overlap values produced by linking the niche of one distribution to a niche generated with random occurrences drawn from the geographic space of the other distribution.

**Results**

|  |  |
| --- | --- |

Fig 1. Thermal tolerance limits inferred for the northern and southern genetic groups.

Species distribution models showed thermal tolerances for the northern genetic group between 3.22ºC and 24.23ºC, and between 1.94ºC and 23.98ºC for southern group (Fig. 1).

Fig 2. Probability of niche overlap between the northern and southern hemisphere distributions (95% credible intervals displayed in dashed lines).

The posterior distribution of the probabilistic niche overlap between the northern and southern hemispheres was 86.88%, while the overlap between the southern and northern hemispheres was 94.59% (Figure 2).

Table 1. Niche similarity tests performed between the northern and southern hemispheres.

|  | D | I |
| --- | --- | --- |
| Observed | 0.550 | 0.705 |
| Equivalency test (p-value) | 1 | 1 |
| Similarity test (p-value) | <0.001 | <0.001 |

The equivalency test failed to reject the null hypothesis (P > 0.01; Table 1); thus, showing the northern and southern hemispheres with identical ecological niches. The similarity test rejected the null hypothesis of the background test, indicating that both niches are more similar than expected from random niches drawn from the available ecological spaces. Together, these results show evidence of niche conservatism between hemispheres in *Macrocystis pyrifera*.

**References**

Swanson, H.K., Lysy, M., POwer, M., Stasko, A.D., Johnson, J.D. & Reist, J.D. (2015) A new probabilistic method for quantifying n-dimensional ecological niches and niche overlap. *Ecology*.

Warren, D.L., Glor, R.E. & Turelli, M. (2008) Environmental niche equivalency versus conservatism: Quantitative approaches to niche evolution. *Evolution*.
