# Supplementary material for: Past climate-driven range shifts structuring intraspecific biodiversity levels of the giant kelp (Macrocystis pyrifera) at global scales
Source: Sci Rep. 2023 Jul 25;13:12046. doi: 10.1038/s41598-023-38944-7 (PMC10368654; doi:10.1038/s41598-023-38944-7)
Supplement: Supplementary file 3 — Supplementary Figures. [file 41598_2023_38944_MOESM3_ESM.docx]

**Supplementary information 3**

**Additional results of genetic structure analyses**

**Additional results of species distribution modelling**

**Ocean currents velocity inferred for the Last Glacial Maximum and the present**

Figure 1. Mean absolute difference of the second order rate of change with respect to the number of genetic groups (Evanno et al., 2005). Analysis performed for the first level of genetic structure, i.e., with all sampled sites.

Figure 2. Mean absolute difference of the second order rate of change with respect to the number of genetic groups (Evanno et al., 2005). Analysis performed for the second level of genetic structure using samples from the northern hemisphere.

Figure 3. Mean absolute difference of the second order rate of change with respect to the number of genetic groups (Evanno et al., 2005). Analysis performed for the second level of genetic structure using samples from the southern hemisphere.

|  |  |
| --- | --- |

Figure 4. Standardized genetic diversity per site as a function of (left and right panels) the natural logarithm of distance to glacial refugia.

|  |  |
| --- | --- |

Figure 5. Standardized genetic diversity per site as a function of distance to putative origin (i.e., Channel Islands, California).

| 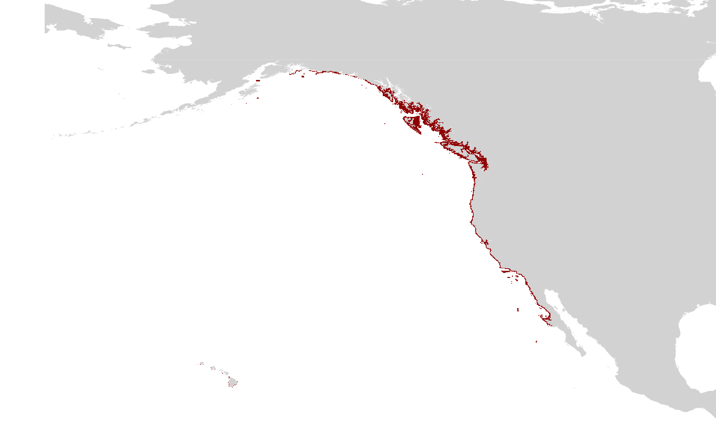 | 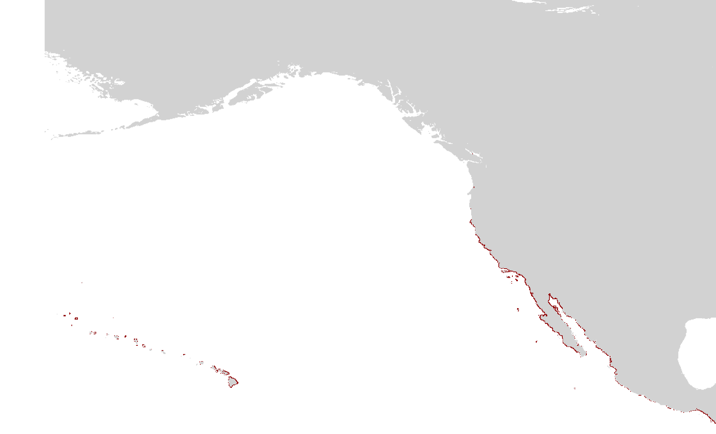 |
| --- | --- |

Figure 6. Potential distribution of *Macrocystis pyrifera* inferred with Species Distribution Modelling for (left panel) the present and (right panel) the Last Glacial Maximum (red color depicting suitable habitats). Figure generated in R computing language (R Foundation for Statistical Computing, 2023; https://www.r-project.org) using the open-source landmass polygon provided by OpenStreetMap (OpenStreet map: User-generated street maps. https://www.openstreetmap.org/).

| 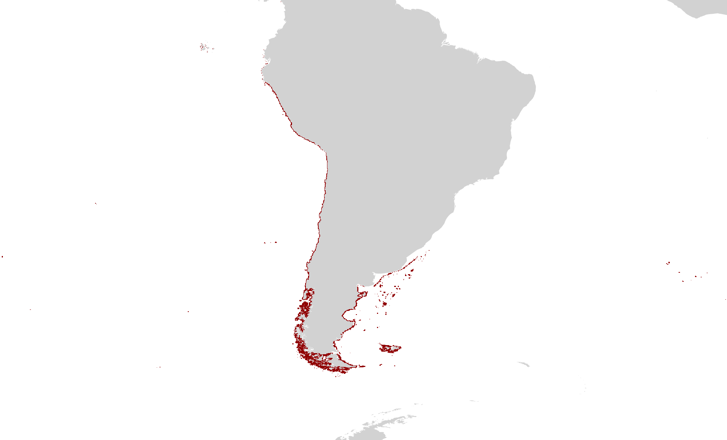 | 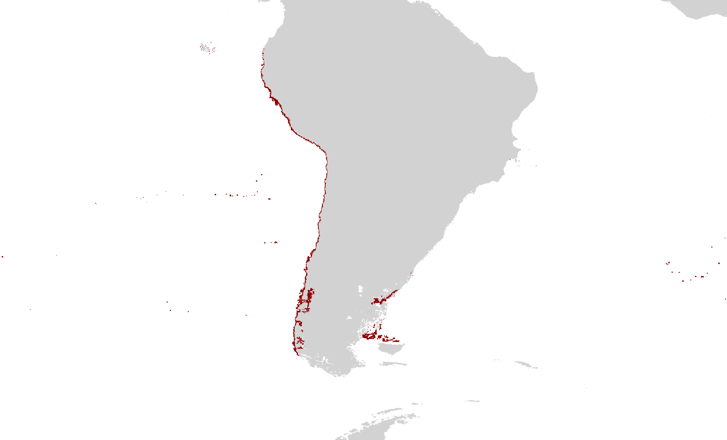 |
| --- | --- |

Figure 7. Potential distribution of *Macrocystis pyrifera* inferred with Species Distribution Modelling for (left panel) the present and (right panel) the Last Glacial Maximum (red color depicting suitable habitats). Figure generated in R computing language (R Foundation for Statistical Computing, 2023; https://www.r-project.org) using the open-source landmass polygon provided by OpenStreetMap (OpenStreet map: User-generated street maps. https://www.openstreetmap.org/).

| 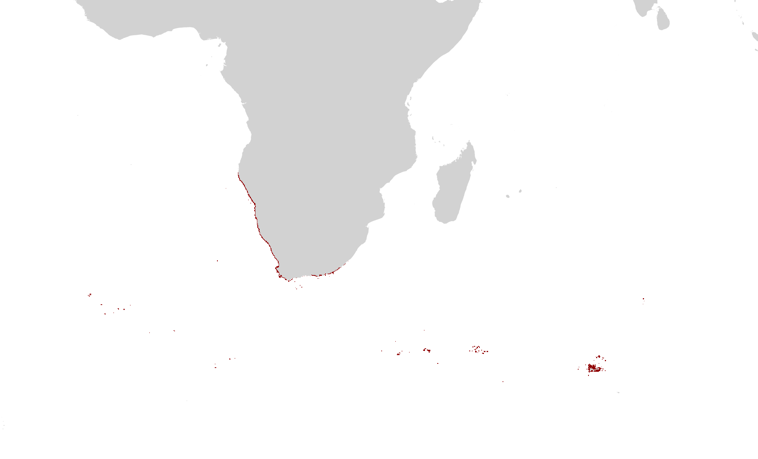 | 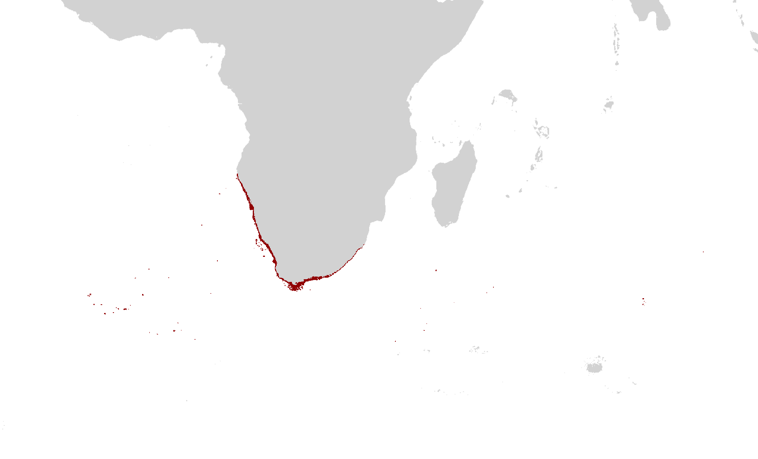 |
| --- | --- |

Figure 8. Potential distribution of *Macrocystis pyrifera* inferred with Species Distribution Modelling for (left panel) the present and (right panel) the Last Glacial Maximum (red color depicting suitable habitats). Figure generated in R computing language (R Foundation for Statistical Computing, 2023; https://www.r-project.org) using the open-source landmass polygon provided by OpenStreetMap (OpenStreet map: User-generated street maps. https://www.openstreetmap.org/).

| 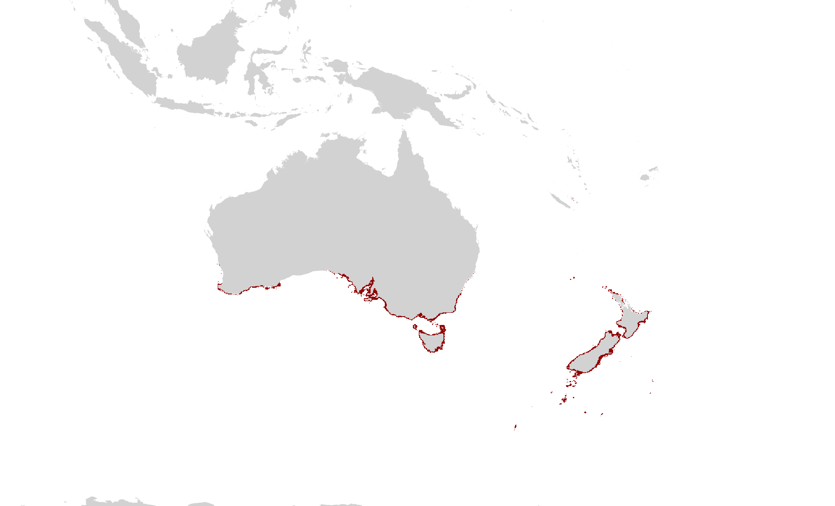 | 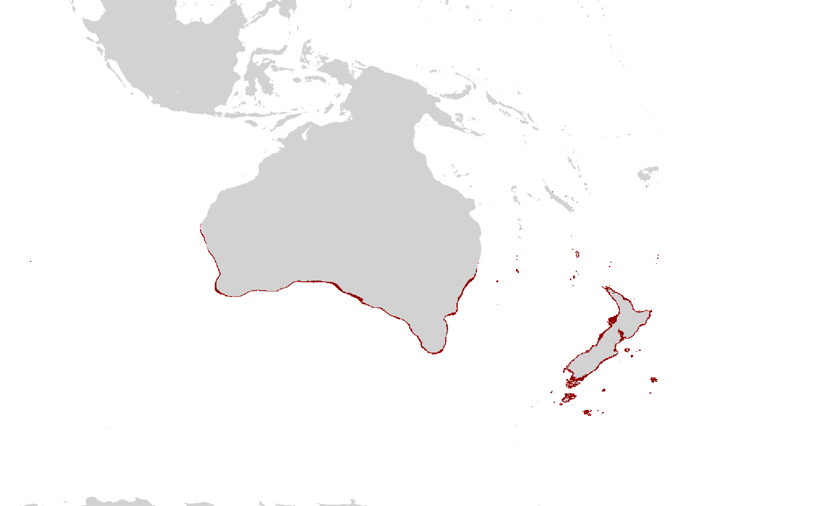 |
| --- | --- |

Figure 9. Potential distribution of *Macrocystis pyrifera* inferred with Species Distribution Modelling for (left panel) the present and (right panel) the Last Glacial Maximum (red color depicting suitable habitats). Figure generated in R computing language (R Foundation for Statistical Computing, 2023; https://www.r-project.org) using the open-source landmass polygon provided by OpenStreetMap (OpenStreet map: User-generated street maps. https://www.openstreetmap.org/).


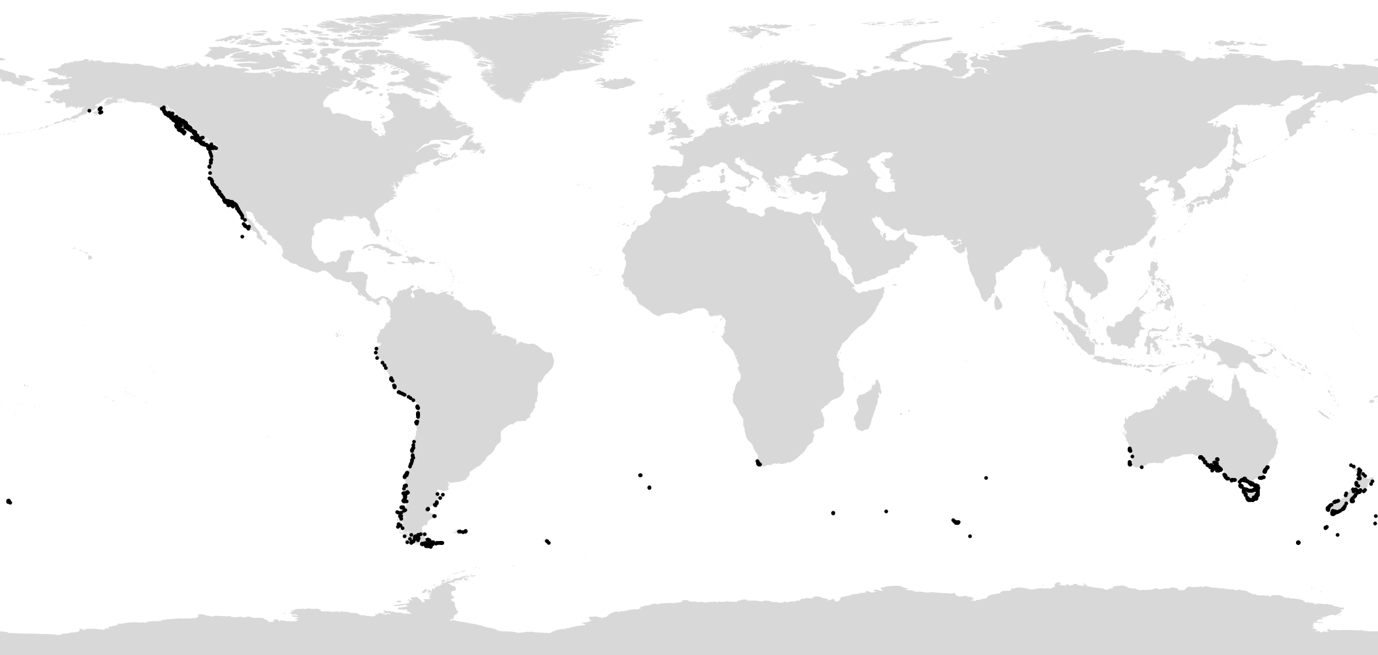


Figure 10. Comprehensive dataset of distribution records of *Macrocystis pyrifera*. Figure generated in R computing language (R Foundation for Statistical Computing, 2023; https://www.r-project.org) using the open-source landmass polygon provided by OpenStreetMap (OpenStreet map: User-generated street maps. https://www.openstreetmap.org/).


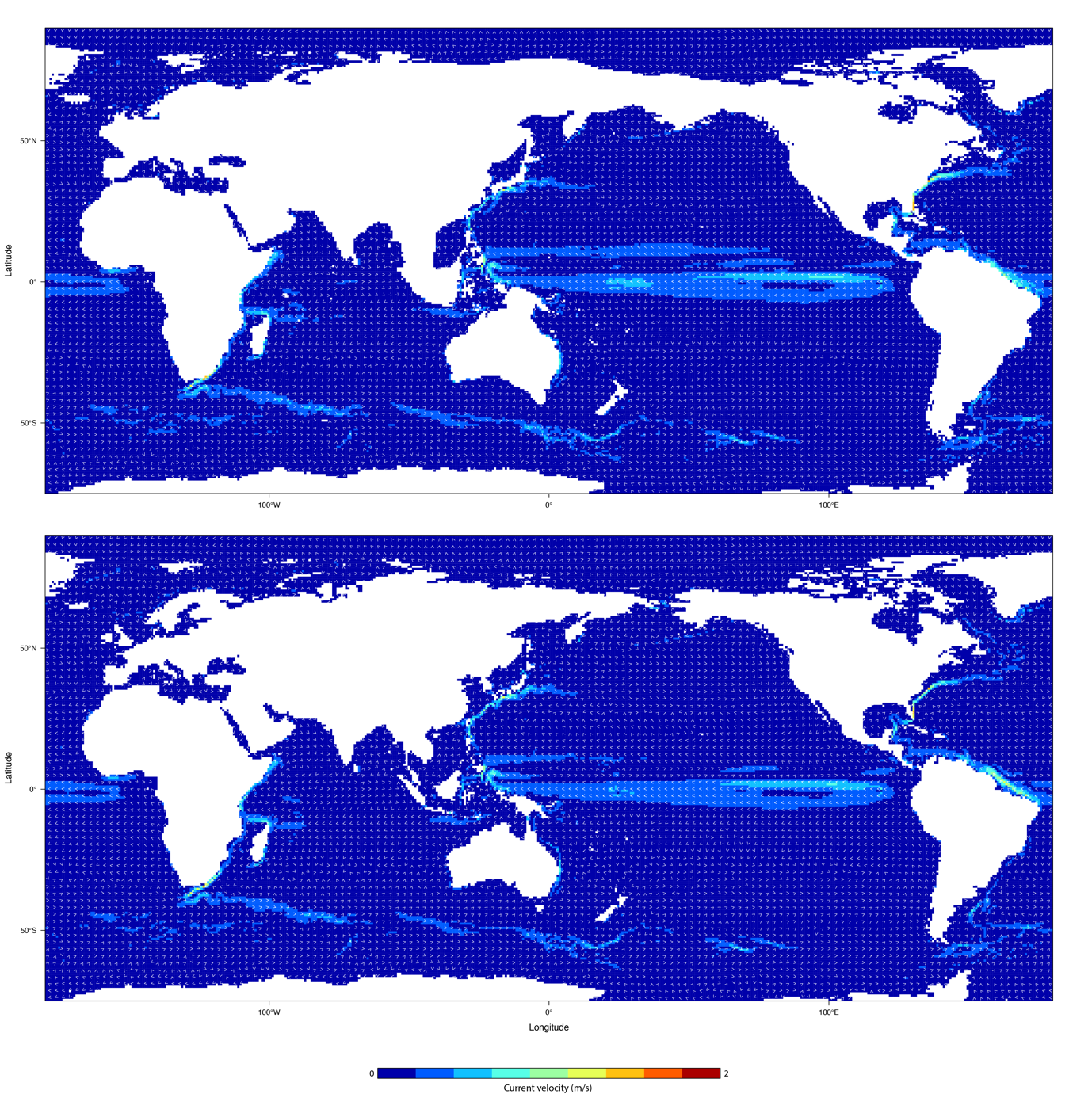


Figure 11. Ocean currents velocity inferred for (top panel) the Last Glacial Maximum and (lower panel) the present. Figure generated in R computing language (R Foundation for Statistical Computing, 2023; https://www.r-project.org) using the data derived from Bio-ORACLE dataset (Assis et al., 2017).

References

Assis, J., Tyberghein, L., Bosch, S., Verbruggen, H., Serrão, E. A., & De Clerck, O. (2017). Bio-ORACLE v2.0: Extending marine data layers for bioclimatic modelling. *Global Ecology and Biogeography*, *27*, 277–284.

De’ath, G. (2007). Boosted trees for ecological modeling and prediction. *Ecology*, *88*, 243–251.

Elith, J., Leathwick, J. R., Hastie, T., & R. Leathwick, J. (2008). Elith, Leathwick & Hastie A working guide to boosted regression trees - Online Appendices Page 1. *Journal of Animal Ecology*, *77*, 802–813.

Evanno, G., Regnaut, S., & Goudet, J. (2005). Detecting the number of clusters of individuals using the software STRUCTURE: A simulation study. *Molecular Ecology*, *14*, 2611–2620.
